# Genome-Wide Architecture of Disease Resistance Genes in Lettuce

**DOI:** 10.1534/g3.115.020818

**Published:** 2015-10-08

**Authors:** Marilena Christopoulou, Sebastian Reyes-Chin Wo, Alex Kozik, Leah K. McHale, Maria-Jose Truco, Tadeusz Wroblewski, Richard W. Michelmore

**Affiliations:** Genome Center and Department of Plant Sciences, University of California, Davis, California 95616

**Keywords:** reverse genetics, NB-LRR, lettuce downy mildew, gene silencing, *Bremia lactucae*

## Abstract

Genome-wide motif searches identified 1134 genes in the lettuce reference genome of cv. Salinas that are potentially involved in pathogen recognition, of which 385 were predicted to encode nucleotide binding-leucine rich repeat receptor (NLR) proteins. Using a maximum-likelihood approach, we grouped the NLRs into 25 multigene families and 17 singletons. Forty-one percent of these NLR-encoding genes belong to three families, the largest being *RGC16* with 62 genes in cv. Salinas. The majority of NLR-encoding genes are located in five major resistance clusters (MRCs) on chromosomes 1, 2, 3, 4, and 8 and cosegregate with multiple disease resistance phenotypes. Most MRCs contain primarily members of a single NLR gene family but a few are more complex. MRC2 spans 73 Mb and contains 61 NLRs of six different gene families that cosegregate with nine disease resistance phenotypes. MRC3, which is 25 Mb, contains 22 *RGC21* genes and colocates with *Dm13*. A library of 33 transgenic RNA interference tester stocks was generated for functional analysis of NLR-encoding genes that cosegregated with disease resistance phenotypes in each of the MRCs. Members of four NLR-encoding families, *RGC1*, *RGC2*, *RGC21*, and *RGC12* were shown to be required for 16 disease resistance phenotypes in lettuce. The general composition of MRCs is conserved across different genotypes; however, the specific repertoire of NLR-encoding genes varied particularly of the rapidly evolving Type I genes. These tester stocks are valuable resources for future analyses of additional resistance phenotypes.

Plants use multiple cell-surface and cytosolic receptors to perceive and react to pathogen attacks. These receptors are categorized into pattern recognition receptors that recognize conserved microbial signatures known as microbe-associated molecular patterns and nucleotide binding-leucine rich repeat receptor (NLR) proteins that recognize pathogen effector molecules (Jones and Dangl 2006; Ronald and Beutler 2010; Monaghan and Zipfel 2012). Most pattern recognition receptors are receptor-like kinases (RLKs) or receptor-like proteins (RLPs), both containing an extracellular domain that can bind ligands and a transmembrane domain (TM) but only RLKs have a cytosolic kinase domain (Monaghan and Zipfel 2012). The majority of the RLKs known to play a role in disease resistance belong to the class of non-arginine-aspartate (non-RD) kinases (Dardick and Ronald 2006; Dardick *et al.* 2012).

NLRs are cytoplasmic receptors that directly or indirectly recognize effectors and activate a strong defense reaction known as effector-triggered immunity (Jones and Dangl 2006; Bozkurt *et al.* 2012). They often contain additional domains but usually not a kinase. NLRs typically consist of a variable N terminus, a conserved internal nucleotide binding (NB)-ARC domain, and a highly polymorphic carboxy (C) terminus with multiple leucine-rich repeats (LRRs) (Hulbert *et al.* 2001). NLRs can be classified into two major groups, TNLs and non-TNLs, based on the presence or absence of a TOLL/interleukin-1 receptor (TIR) domain at the N-terminus (Takken and Goverse 2012). The sequence conservation of the NB domain within and between species is high and allows the classification into TNL *vs.* non-TNL proteins (Meyers *et al.* 2003; McHale *et al.* 2006). Some non-TNLs contain a coiled-coil (CC) domain and therefore collectively non-TNLs are referred to as CNLs.

The NLR-encoding genes comprise one of the most polymorphic and abundant gene super-families in plants (Clark *et al.* 2007; Karasov *et al.* 2014). The numbers of NLR-encoding genes vary greatly among species, for example, there are only 54 in *Carica papaya* (Porter *et al.* 2009), 150 in *Arabidopsis thaliana* (Meyers *et al.* 2003), 571 in *Medicago truncatula* (Shao *et al.* 2014), 465 in soybean (Shao *et al.* 2014), 402 in *Populus trichocarpa* (Kohler *et al.* 2008), 535 in *Oryza sativa* (Zhou *et al.* 2004), and as many as 1015 in *Malus domestica* (Arya *et al.* 2014). The prevalence of the different classes also can vary considerably; TNL families have expanded in eudicots but are absent in grass genomes (Meyers *et al.* 2005; Yue *et al.* 2012). NLR-encoding genes are clustered in the genome as a result of lineage-specific segmental duplication events followed by local rearrangements (Zhang *et al.* 2014). Expansion of individual clusters occurs through tandem duplications resulting from unequal crossing over (Richly *et al.* 2002; Meyers *et al.* 2003; Zhou *et al.* 2004). The complexity of these clusters also is affected by gene loss through natural selection acting on a birth-and-death process (Michelmore and Meyers 1998; Leister 2004; Mondragon-Palomino and Gaut 2005; Guo *et al.* 2011). NLR-encoding genes also exhibit presence/absence variation polymorphisms within or among related species (Kuang *et al.* 2004; Shen *et al.* 2006; Guo *et al.* 2011; Luo *et al.* 2012). These genetic and evolutionary processes have resulted in complex, variable clusters of NLR genes in the genome that confer resistance to multiple diverse pathogens.

Lettuce (*Lactuca sativa*) is one of the most valuable vegetable crops in the United States, with a value of approximately 2 billion dollars *per annum* and is host to a wide range of pathogens and pests. A wealth of genetic and genomic resources and tools are now available that enable genetic and functional studies of disease resistance in lettuce. More than 52 loci have been genetically defined that confer resistance phenotypes to 10 pathogens and one pest, nearly all of which cosegregate with clusters of NLR-encoding genes (McHale *et al.* 2009; Hayes *et al.* 2011; M. J. Truco and R. W. Michelmore, unpublished data). Lettuce downy mildew, caused by the obligate biotroph *Bremia lactucae* Regel, is the most economically important disease affecting the lettuce production worldwide (Davis *et al.* 1997) that has been studied extensively (Michelmore and Wong 2008). Lettuce downy mildew is one of the most characterized gene-for-gene plant−pathogen interactions; more than 25 genes for resistance to downy mildew (*Dm*) have been identified, most of which are dominant genes that confer resistance in a gene-for-gene manner (Crute and Johnson 1976; Farrara *et al.* 1987; Ilott *et al.* 1989; Michelmore *et al.* 2009). Many of these resistance genes have been introgressed from wild *Lactuca* species. Studies of sequence diversity between lettuce cultivars showed elevated levels of polymorphism for five major resistance clusters (MRCs) on chromosomes 1, 2, 3, 4, and 8 (McHale *et al.* 2009; Truco *et al.* 2013). A draft 2.4-Gb reference sequence assembly is now available for the 2.7-Gb genome of *L. sativa* cv. Salinas along with an ultradense genetic map that assigns more than 96% of the assembled sequence to 1460 genetic bins ordered along the nine chromosomal linkage groups (http://lgr.genomecenter.ucdavis.edu; S. Reyes-Chin Wo, A. Kozik, D. Lavelle, and R. W. Michelmore, unpublished data).

The MRC on chromosome 2 is the most studied MRC in lettuce. MRC2 includes at least eight *Dm* genes (McHale *et al.* 2009), including *Dm3* the first resistance (*R*) gene cloned from lettuce (Shen *et al.* 2002), as well as resistance to root aphid (*Ra*) (Ellis *et al.* 2002) and a quantitative trait locus (QTL) for resistance to anthracnose (*ANT1*) (McHale *et al.* 2009). Analysis of deletion mutants during the map-based cloning efforts to clone *Dm3* identified 24 *Resistance Gene Candidate 2* (*RGC2*) family members, most of which mapped within MRC2, spanning a region of at least 3 Mb in cv. Diana (Meyers *et al.* 1998a). Sequencing of *dm3* mutants and transgenic complementation established *RGC2B* as the gene conferring *Dm3* (Shen *et al.* 2002). Genetic analysis revealed that this region was not highly recombinogenic but subject to gene conversions and spontaneous mutations associated with chromosomal deletions (Chin *et al.* 2001). Analysis of *RGC2* sequences from 47 accessions of *Lactuca* spp. indicated that gene conversions, mutation, and recombination events have been involved in the complex evolutionary history of this locus and distinguished two types of *RGC2* genes: fast-evolving Type I *RGC2* genes that are the product of frequent sequence exchange, are under diversifying selection, and exhibit low prevalence among different lettuce genotypes and slowly evolving Type II *RGC2* genes that have maintained allelic/orthologous relationships and are under balancing selection (Kuang *et al.* 2004). A fragment of the LRR-encoding sequence of *RGC2B* used as the trigger sequence for RNA interference (RNAi) abrogated the resistance mediated by not only *Dm3* but also *Dm14*, *Dm16*, *Dm18*, and *Ra*, suggesting that these linked resistance phenotypes also are encoded by *RGC2* family members (Wroblewski *et al.* 2007).

In this paper we use a combination of genetic, whole-genome, and functional genomic approaches to determine the genome-wide distribution, sequence relationships, and phenotypes of NLR-encoding genes in lettuce. Candidate *R* genes were identified with motif searches and a maximum-likelihood approach was used to group NLR-encoding genes into 42 families. The lettuce reference genome assembly allowed the analysis of the five MRCs in lettuce. MRC2, MRC3, and MRC8 are the focus of this paper. MRC1 and MRC4 have been described in detail previously (Christopoulou *et al.* 2015). A library of 33 RNAi tester stocks was generated and analyzed for members of 12 *RGC* families; it was used to identify four families involved in 16 resistance specificities against two pathogens. This set of tester stocks is a valuable resource for future analysis of additional resistance phenotypes.

## Materials and Methods

### Plant materials and DNA isolation

All lettuce accessions were obtained from our collection of germplasm at UC Davis. Plants were grown under greenhouse conditions. DNA isolations were performed on seedlings or leaf tissue with a CTAB protocol (Bernatzky and Tanksley 1986) with minor modifications.

### Generation and evaluation of transgenic RNAi tester stocks

Candidate genes were selected for RNAi analysis based on their cosegregation with disease resistance phenotypes and representation of as many diverse gene sequences as possible. This study was initiated before the sequencing and assembly of the lettuce genome. Thus, the majority of candidate genes could not be assigned initially to a particular family due to limited sequence available from the lettuce EST collection; the average length of the cDNA clones was 0.8 kb and the maximum 2 kb. Motif searches of the lettuce ESTs and genomic amplification using degenerate primers were used to generate a library of candidate NLR encoding genes (McHale *et al.* 2009). Only sequences that colocalized with mapped resistance specificities were considered as candidates for silencing. The most divergent genes, based on the available sequenced fragments, were selected for RNAi, including at least one candidate RNAi construct for every cluster of mapped resistance specificities.

Selected EST clones were obtained from the Arizona Genomics Institute (http://www.genome.arizona.edu/) and used as a template for amplification of RNAi trigger sequences for each gene (supporting information, Table S1). iProof high-fidelity DNA polymerase was used for all DNA amplifications according to manufacturer’s instructions (Bio-Rad Laboratories, Hercules CA). Sequences were validated by Sanger sequencing. One 400- to 500-bp fragment of each candidate gene (Table 1) was cloned as inverted repeats into either the pGollum or pSmeagol vectors. The first set of constructs used pGollum (Wroblewski *et al.* 2007, 2014) and resulted in the trigger sequence between a 400-bp fragment of the *UidA*, a beta-glucuronidase (GUS) encoding reporter gene and the PDK intron in an inverted repeat structure (Figure S1). The later set of constructs was made with pSmeagol by the use of the same restriction sites as in pGollum (Figure S1). Initial RNAi constructs were generated with pGollum, and later constructs were generated with pSmeagol because data indicated that the arrangement in pSmeagol (Figure S1) provided greater levels of RNAi (Wroblewski *et al.* 2014); however, RNAi was successfully induced with both versions.

**Table 1:** All RNAi constructs generated and tested targeting NLR-encoding genes, the gene model of the progenitor EST sequences were derived from, gene family they belong to, resistance specificities tested, vector used, predicted number of *RGCs* targeted for RNAi, and resistance phenotypes abrogated

RNAi construct (Chromosome*^a^*)	Similar Salinas Gene Model (% Identity), NLR Family	Number of *RGC* Members Predicted to be Targeted by RNAi/Total Number of *RGC* Members	*Dm* Specificities Tested*^b^*	Effectors tested by Agro-Infiltration	Silenced Phenotypes*^b^*	Vector
QGC20G02_LRR_RNAi*^d^* (chr 1)	Lsa038417.1 (99.7%), not assigned	N/A*^c^*	*Dm17*, *Dm43*			pGollum
QGD13H21_NB_RNAi*^d^* (chr 1)	Lsa139090.1 (87.6%), *RGC16*	29/62 *RGCC16*	*Dm17*, *Dm43*			pGollum
QGD7B12_LRR_RNAi*^d^* (chr1)	Lsa037789.1 (99.8%), *RGC34*	1/4 *RGC34*	*Dm10*, *Dm43*, *Dm5/8*, *Dm17*, *Dm45*, *Dm36*	AvrB, Avrpm1, AvrRpt2		pGollum
CLV_S1_Contig142_NB_RNAi*^d^* (chr 1)	Lsa053434.1 (91.1%), *RGC1*	18/22 *RGC1*	*Dm10*, *Dm43*, *Dm5/8*, *Dm17*, *Dm45*, *Dm36*	AvrB, Avrpm1, AvrRpt2		pGollum
QGF16M04_LRR_RNAi*^d^* (chr 1)	Lsa039421.1 (88%), *RGC1*	15/22 *RGC1*	*Dm10*, *Dm43*, *Dm5/8*, *Dm17*, *Dm45*, *Dm36*	AvrB, Avrpm1, AvrRpt2	*Dm5/8*, *Dm45*, *HR-AvrB*, *HR-AvrRpm1*, *HR-AvrRpt2*	pGollum
QGF20G21_LRR_RNAi*^d^* (chr 1)	Lsa025432.1 (96%), *RGC1*	16/22 *RGC1*	*Dm10*, *Dm43*, *Dm5/8*, *Dm17*, *Dm45*, *Dm36*	AvrB, Avrpm1, AvrRpt2	*Dm5/8*	pGollum
		4/16 *RGC21*				
QGD6G21_NB_RNAi*^d^* (chr 1)	Lsa002259.1 (84.1%), *RGC16*	19/62 *RGC16*	*Dm10*, *Dm43*, *Dm5/8*, *Dm17*, *Dm45*, *Dm36*	AvrB, Avrpm1, AvrRpt2		pGollum
QGC12K15_TIR_RNAi*^d^* (chr 1)	Lsa031265.1 (100%), *RGC16*	20/62 *RGC16*	*Dm10*, *Dm43*, *Dm5/8*, *Dm17*, *Dm45*, *Dm36*	AvrB, Avrpm1, AvrRpt2		pGollum
CLRX1526_LRR_RNAi*^d^* (chr 1)	Lsa004568.1 (98.5%), *RGC16*	28/62 *RGC16*	*Dm10*, *Dm43*, *Dm5/8*, *Dm17*, *Dm45*, *Dm36*	AvrB, Avrpm1, AvrRpt2		pGollum
CLRX7678_NB_RNAi*^d^* (chr 1)	Lsa002271.1 (97.8%), *RGC16*	35/62 *RGC16*	*Dm10*, *Dm43*, *Dm5/8*, *Dm17*, *Dm45*, *Dm36*	AvrB, Avrpm1, AvrRpt2		pGollum
CLSX3769_NB_RNAi*^d^* (chr 1)	Lsa021698.1 (99.3%), *RGC16*	21/62 *RGC16*	*Dm10*, *Dm43*, *Dm5/8*, *Dm17*, *Dm45*, *Dm36*	AvrB, Avrpm1, AvrRpt2		pGollum
CLSM10181_NB_RNAi*^d^* (chr 1)	Lsa029824.1 (89.7%), *RGC2*	14/20 *RGC2*	*Dm10*, *Dm43*, *Dm5/8*, *Dm17*, *Dm45*, *Dm36*	AvrB, Avrpm1, AvrRpt2		pGollum
LserNBS03_NB_RNAi*^d^* (chr 1)	Lsa021288.1 (100%), *RGC21*	15/28 *RGC21* 4/22 *RGC1*	*Dm10*, *Dm43*, *Dm5/8*, *Dm17*, *Dm45*, *Dm36*	AvrB, Avrpm1, AvrRpt2		pGollum
Contig_1306_NB_RNAi*^d^* (chr 1)	Lsa014706.1 (99%), *RGC6*	2/2 *RGC6*	*Dm10*, *Dm43*, *Dm5/8*, *Dm17*, *Dm45*, *Dm36*	AvrB, Avrpm1, AvrRpt2		pSmeagol
Contig_7390_LRR_RNAi*^d^* (chr 1)	Lsa017144.3 (98.87%), not assigned	N/A^c^	*Dm10*, *Dm43*, *Dm5/8*, *Dm17*, *Dm45*, *Dm36*	AvrB, Avrpm1, AvrRpt2		pGollum
AY153836_NB_RNAi*^d^* (chr 1)	Lsa031651.1 (100%), *RGC16*	20/62 *RGC16*				pGollum
CLSS6596_TIR_RNAi*^d^* (chr 4)	Lsa011787.1 (94.6%), *RGC12*	27/55 *RGC12*	*Dm4*, *Dm7*, *Dm11*, *Dm44,*		*Dm4*, *Dm11*, *Dm44*	pGollum
1/62 *RGC16*
CLSM18700_LRR_RNAi*^d^* (chr 4)	Lsa025738.1 (99.4%), *RGC12*	28/55 *RGC12*	*Dm4*, *Dm7*, *Dm11*, *Dm44,*			pGollum
		2/6 *RGC20*				
CLSX586_NB_RNAi***^d^*** (chr 4)	Lsa007832.3 (99.9%), *RGC12*	39/55 *RGC12*	*Dm4*, *Dm7*, *Dm11*, *Dm44*, *Dm5/8*, *Dm3*		*Dm4*, *Dm7*, *Dm11*, *Dm44*	pGollum
1/6 *RGC20*
LsatNBS09_NB_RNAi (chr 2)	Lsa125929.1 (100%), *RGC4*	31/42 *RGC4*	*Dm1*			pSmeagol
RGC2B_LRR_RNAi*^e^* (chr 2)	*RGC2B*	11/20 *RGC2*	*Dm1*, *Dm3*, *Dm2*, *Dm6*, *Dm14*, *Dm15*, *Dm16*, *Dm18*, *Ra*		*Dm3*, *Dm14*, *Dm16*, *Dm18*, *Ra*	pGollum
RGC2B_NB_RNAi (chr 2)	*RGC2B*	12/20 *RGC2*	*Dm2*, *Dm3*, *Dm6*, *Dm15*, *Dm16*, *Dm18*		*Dm3*, *Dm6*, *Dm18*	pGollum
LsatNBS05_NB_RNAic (chr 2)	Lsa009249.1 (99.32%), RGC18	27/29 RGC18	No specificities tested			pGollum
QGC7A16_LRR_RNAi (chr 3)	Lsa021288.1 (99.84%), *RGC21*	15/28 *RGC21*	*Dm13*		*Dm13*	pSmeagol
AF017754_NB_RNAi (chr 3)	Lsa034516.1 (98.73%), *RGC4*	26/42 *RGC4*	*Dm13*			pSmeagol
LserNBS02_NB_RNAi (chr 3)	Lsa002429.1 (98.84%), *RGC21*	20/28 *RGC21*	*Dm13*			pSmeagol
AY153833.1_LRR_RNAi (chr 8)	Lsa010510.1 (99.65%), *RGC15*	6/11 *RGC15*	*Dm5/8*	AvrRps4		pSmeagol
LE0395_LRR_RNAi (chr 8)	Lsa022448.1 (86.05%), *RGC29*	4/5 *RGC29*	*Dm5/8*	AvrRps4		pGollum
LEO414_LRR_RNAi (chr 8)	Lsa031490.1 (100%), *RGC9*	1/4 *RGC7*		AvrRps4		pGollum
LEO266_TIR_RNAi (chr 8)	Lsa017362.1 (97.6%), *RGC4*	6/42 *RGC4*		AvrRps4		pGollum
Lsat11_NB_RNAi (chr 8)	Lsa088367.1 (99.49), *RGC34*	3/3 *RGC34*		AvrPpiC		pGollum
Contig5632_TIR_RNAi (chr 8)	Lsa022918.1 (85.28%), *RGC4*	30/42 *RGC4*		AvrPpiC		pSmeagol
QGD14O14_NB_RNAi (chr 8)	Lsa083425.1 (89.1%), *RGC5*	1/1 *RGC5*		AvrPpiC		pSmeagol

RNAi, RNA interference; EST, expressed sequence tag; NLR, nucleotide binding-leucine rich repeat receptor; HR, hypersensitive response; N/A, not applicable.

a Chromosome on which the phenotypes tested map to.

b Genes expressed in wild-type germplasm and evaluated for abrogration by RNAi construct in stable transgenics.

c Targeted NLR encoding gene cosegregates with resistance to lettuce dieback.

d Described in Christopoulou *et al.* 2015.

e Described in Wroblewski *et al.* 2007.

Validated clones were transferred to *Agrobacterium tumefaciens* strain LBA4404 (Hoekema *et al.* 1983) by electroporation (Dower *et al.* 1988). Stable transgenics of cv. Cobham Green (that lacks any of the tested disease resistance phenotypes) expressing each RNAi construct were generated by cocultivation with *A. tumefaciens* by the U.C. Davis Ralph Pearson Transformation Facility (http://ucdptf.ucdavis.edu/; Michelmore *et al.* 1987)). Typically 10−15 primary (T_1_) transgenic lines were generated and evaluated for each construct.

Efficacy of silencing was evaluated in transgenic T_1_ plants and their progeny by the level of silencing of the *UidA* gene, which is simultaneously targeted for RNAi by these constructs as described previously (Wroblewski *et al.* 2005, 2007). GUS expression levels were determined using *Agrobacterium*-mediated transient expression assays (Schöb *et al.* 1997). Two leaves were infiltrated for each GUS assay and the assays were repeated at least twice (Christopoulou *et al.* 2015). The presence of the RNAi transgene *in planta* was confirmed by polymerase chain reaction (PCR) using primers *TrangeneF* and *TransgeneR*, which amplify a ∼800-bp fragment spanning one half of the inverted repeat structure (Table S1). Primers *Le9005L* and *Le9005R* were used as a control to confirm successful PCR amplifications in progeny segregating for the RNAi transgene.

### Phenotypic evaluation of RNAi transgenics

**Figure 1:**
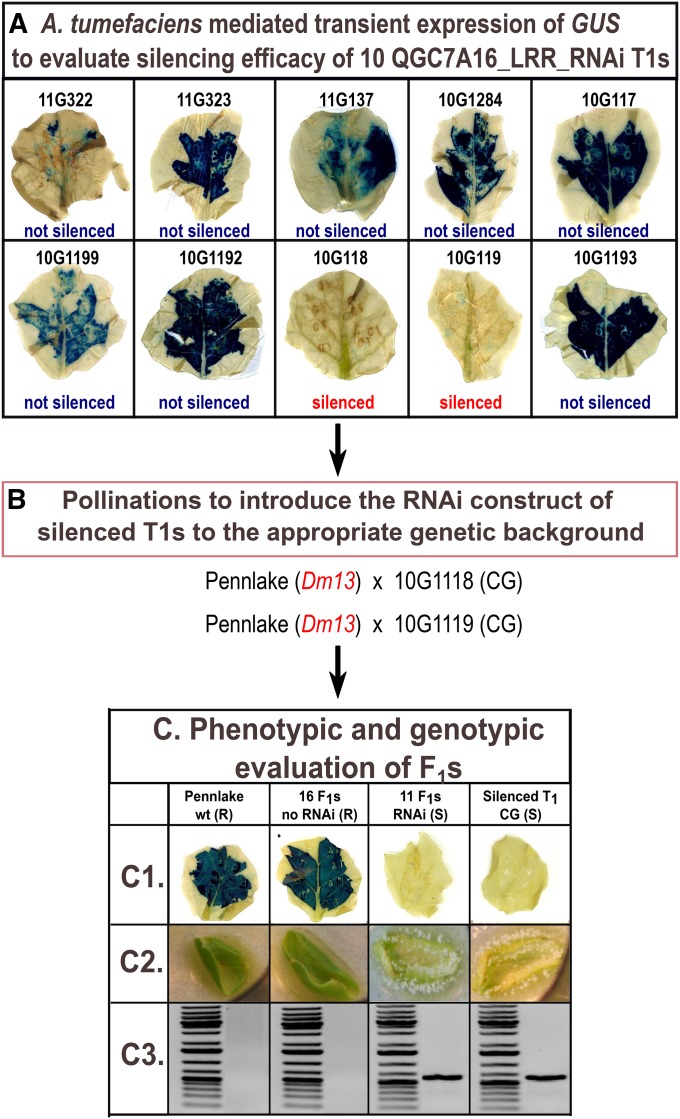
Flowchart of the experimental design for the evaluation of RNA interference (RNAi) constructs, in particular QGC7A16_LRR_RNAi. The methodology applies to all constructs described in this study (different genetic background and isolates used in each case, as described in Table 1 and Table 2). (A) Transient beta-glucuronidase (GUS) expression assays were used to evaluate silencing efficiency of primary transgenic lines (T_1_s). (B) The most efficiently silenced T_1_s were crossed to cv. Pennlake, carrying *Dm13*. (C) Fifteen and ten F_1_s derived from each cross were evaluated for silencing efficiency [GUS assays and (PCR) polymerase chain reaction detection] and resistance to *B. lactucae* isolate CS12. Pathogenicity assays were performed on detached cotyledons and repeated on leaf disks of 3- to 4-week-old plants. Sporulation was only observed in the presence of the RNAi construct (C2). The presence of the transgene was detected with GUS transient assays (C1) and further confirmed by polymerase chain reaction detection (C3).

T_1_ lines lacking *GUS* expression, and therefore presumed to be silenced for the targeted NLR-encoding gene, were selected for assays of resistance. Two or three independent silenced T_1_ lines per construct were crossed to different genotypes carrying the resistance phenotypes to be tested. The F_1_s were tested for DM resistance and/or hypersensitive response (HR) elicited by bacterial effectors as well as for silencing of GUS expression (Table 1). The F_1_s were expected to segregate for the silencing phenotype because the T_1_ transgenics were hemizygous. On average 10 to 15 F_1_s, were tested per cross for each resistance phenotype (Figure 1). Resistance to DM was evaluated with *B. lactucae* isolates diagnostic for specific *Dm* genes (Table 2) on detached cotyledons and detached leaves as described previously (Christopoulou *et al.* 2015). To evaluate HR to bacterial effectors, F_1_s at the four- to six-leaf stage were pressure infiltrated with *A. tumefaciens* strain C58 expressing AvrB, AvrRpm1, AvrRpt2, AvrRps4, or AvrPpiC as described previously (Christopoulou *et al.* 2015). A C58 strain expressing AvrPto was used as a positive control and PTFS40 strain expressing *UidA* as the negative control.

**Table 2:** Lettuce genotypes, their respective resistance specificities, and the diagnostic *B. lactucae* isolates used to test for LDM resistance

Lettuce Cultivars or Breeding Lines	Resistance Specificity	HR to Bacterial Effectors	Isolates Tested (Relevant Avr)
Capitan	*Dm11*		CG1 (Avr11)
CGN14263	*Dm43*, *Dm44*		C01O879 (Avr43)
LSE57/15	*Dm7*		R60 (Avr7)
R4T57	*Dm4*		C980648ED (Avr4)
Diana	*Dm1*, *Dm3*, *Dm5/8*, *Dm7*		IM25P11 (Avr3)
			CG1 (Avr5/8),
			R60 (Avr7)
Ninja	*Dm3*, *Dm11*, *Dm36*	AvrB, AvrRpm1, AvrRpt2	CG1 (Avr36)
Valmaine	*Dm5/8*, *Tu*, *Fusarium*	AvrPpiC, AvrRps4	CG1 (Avr5/8)
UCDM10	*Dm10*		C83M47 (Avr10)
LSE102	*Dm17*		C01O879 (Avr17)
09G1126	*Dm43*		C01O879 (Avr43)
09G952	*Dm45*		C01O879 (Avr45)
Pennlake	*Dm13*		CS12 (Avr13)
Cobham Green	None known		


LDM, lettuce downy mildew; HR, hypersensitive response.

### *In silico* identification of RNAi targets

BLAST searches (Altschul *et al.* 1997) using the fragment of the gene that had been cloned as an inverted repeat were performed to identify potential targets of the trigger sequences in the lettuce genome. The minimum requirement for a target was one or more contiguous sequences of at least 21 nucleotides with 100% identity to the reference genome of cv. Salinas.

### Identification and classification of genes involved in pathogen recognition

Predicted gene models of the lettuce reference genome *L. sativa* cv. Salinas (http://lgr.genomecenter.ucdavis.edu; R. W. Michelmore and S. Reyes-Chin Wo, unpublished data) were screened using Hidden Markov Models (HMMs), using *E*-value 1 e^−10^ as the cutoff, to search for genes encoding domains characteristic of NLR, RLK, and ABC transporter proteins that are known to be involved in pathogen recognition in other plant species. The HMMs (acquired from http://pfam.xfam.org/ unless indicated otherwise) were PF00931.17 and NBS_712.hmm (HMM profile available at http://niblrrs.ucdavis.edu/At_RGenes/) for the NB domain, PF01582.15 for TIR, 9 HMMS for the LRR (PF00560.28, PF07723.8, PF007725.7, PF12799.2, PF13306.1, PF13516.1, PF13504.1, PF13855.1 and PF14580.1), PF00069.20 for protein kinase, PF00005.22 for ABC transporters, and PF00400 for the WD40 domain. The CC motifs were predicted with Paircoil2 at *P* scores below 0.025 (McDonnell *et al.* 2006). TM domains were predicted using both TMHMM 2.0 (Krogh *et al.* 2001) and SCAMPI (Bernsel *et al.* 2008). Gene models with protein kinase, TM and LRR domains were classified as putative RLK encoding genes. The presence of an LRR domain alone classified genes in a separate category since LRR motifs can be found in many functionally unrelated proteins that are not involved in disease resistance (Kobe and Deisenhofer 1994).

A few additional genes with domains characteristic of NLR- and RLP-encoding genes were identified, using InterproScan, when an improved genome annotation of the reference genome of cv. Salinas became available at the end of the study. Twenty-eight additional gene models (Table S2) predicted to have a NB domain were combined with the results of the HMM searches to generate the final list of predicted gene models involved in pathogen recognition.

### Cladistic analysis of NLR-encoding genes

For analysis of the sequences of putative NLR-encoding genes, the NB domain was extracted with HMMER3 (http://hmmer.org/.) using default parameters with NBS_712.hmm. The amino acid sequences were trimmed to ∼30 aa before the Walker A and 10 aa after the MHD motifs. Multiple-sequence alignments were generated with MAFFT (version 7.1, einsi parameters, 1,00 maximum iterations) (Katoh and Standley 2013) and refined manually with the alignment editor GeneDoc (Nicholas *et al.* 1997). The sequence identity was estimated by pairwise comparisons by the use of ClustalX over the entire alignment. Maximum-likelihood analyses were conducted with the customizable version of RAxML 8.0 (Stamatakis 2014), hosted on the CIPRES portal (Miller *et al.* 2010). The Jones-Taylor-Thornton protein evolutionary model with a GAMMA model of heterogeneity and empirical amino acid frequencies was selected as the best-fitting substitution matrix based on ProtTest 3.3 (Darriba *et al.* 2011). Branch support values were calculated by 1000 replicates of fast bootstrapping (Stamatakis *et al.* 2008). Genes were assigned to families using a cutoff of 70% nucleotide sequence identity. We used Dendroscope (Huson *et al.* 2007) to display the sequence relationships of the *RGC*s.

### Identification of RGC2 sequences in cv. Salinas

Sequencing and assembly of the lettuce reference genome permitted a detailed comparison of the MRC2 structure in cv. Salinas with that previously deduced for cv. Diana (Meyers *et al.* 1998a). The sequences of 22 *RGC2* members obtained from cv. Diana (Meyers *et al.* 1998a) were searched by BLASTN against the cv. Salinas genome assembly. Presence/absence was determined by reciprocal best BLAST hits at a minimum of 97% identity and 97% of the query represented in the alignment. The lack of a unique best match, in terms of both sequence identity and length of alignment, was recorded as absence of that particular family member in the Salinas genome. To address possibility of miss-assembly, the raw genomic reads from cv. Salinas were mapped back to the query sequences using the CLC Genomics Workbench (http://www.clcbio.com), using parameters: mismatch cost = 2, insertion and deletion cost = 3, minimum length fraction = 0.9 and similarity 1. The genome assembly and the raw reads of cv. Diana were used as a control.

### Identification of singleton NLR-encoding genes in other lettuce genotypes

The draft genome assemblies of lettuce cvs. Diana, Valmaine, Greenlake, La Brillante, and PI125246 of *L. sativa* and accession US96UC23 of the wild progenitor *L. serriola* (D. Lavelle and R. W. Michelmore, unpublished data) were mined for the presence of singleton NLR encoding genes. The identification of the best BLAST hits was performed as described in the section “Identification of *RGC2* sequences in cv. Salinas.”

#### Statement on data and reagent availability:

All vectors, silencing constructs, and *B. lactucae* isolates are available upon request. File S1 describes the cloning process for the RNAi vector. Figure S1 displays the orientation of the inverted repeats in both RNAi vectors. Figure S2 shows the distribution of NLR and non-NLR encoding genes as well as the position of the scaffolds and the genetic bins of MRC2 on chromosome 2. Table S1 has all the primer sequences. Table S2 lists all the genes putatively involved in pathogen recognition ordered on chromosomal linkage groups along with all the mapped disease resistance phenotypes. Table S3 shows all the NLR encoding genes predicted to be targeted by the RNAi constructs tested for the resistance phenotypes in MRC8A. Table S4 has all the predicted RNAi targets for LEO266_TIR_RNAi. Table S5 lists all the genes predicted to be silenced by constructs QGC7A16_LRR_RNAi and LserNBS02_NB_RNAi.

### Data availability

The lettuce genome sequence is available at https://lgr.genomecenter.ucdavis.edu/. The genome sequences also can be found at GenBank: http://www.ncbi.nlm.nih.gov/bioproject/68025 and http://www.ncbi.nlm.nih.gov/genome/352/.

## Results

### Genome-wide identification of candidate resistance genes

Motif searches and InterproScan identified 1134 gene models in the reference genome of lettuce cv. Salinas that encoded domains characteristic of proteins involved in pathogen recognition (Table S2). Although 408 gene models were designated initially as NLRs, 23 were classified as genes encoding only LRRs because the NB motifs could not be validated visually. A total of 385 genes were used to generate a multiple sequence alignment of the NB domain for cluster analysis. N- and C-terminal domains were identified for 328 of 385 genes; the remaining 57 lacked one or more characteristic domains, which could reflect the diversity of gene structures present and/or be an artifact of assembling multi-gene families from short reads (Table 3). Forty-five genes were predicted to have a TIR domain but not an NB or LRR. The average length of the coding region for genes containing at least a NB domain (385 in total) was 3064 kb.

**Table 3:** Classification of the 1134 genes identified with domains characteristic of *R* genes

Predicted Protein Domains	No. Genes	Acronym
CC, NB, LRR	47	CNL
TIR, NB, LRR	189	TNL
NB, LRR (CNL-type)	78	NcL
NB, LRR (TNL-type)	3	NtL
NB (CNL-type)	40	Nc
NB (TNL-type)	17	Nt
CC, NB	2	CN
TIR, NB	9	TN
TIR	45	T
LRR	248	L
TM, LRR	155	RLP
P kinase, LRR	28	PkinL
P kinase, TM, LRR	270	RLK
P kinase, TIR	1	PkinT
WD40, LRR	2	

CC, coiled-coil; NB, nucleotide binding; LRR, leucine-rich repeat; TIR, TOLL/interleukin-1 receptor; TM, transmembrane.

**Figure 2:**
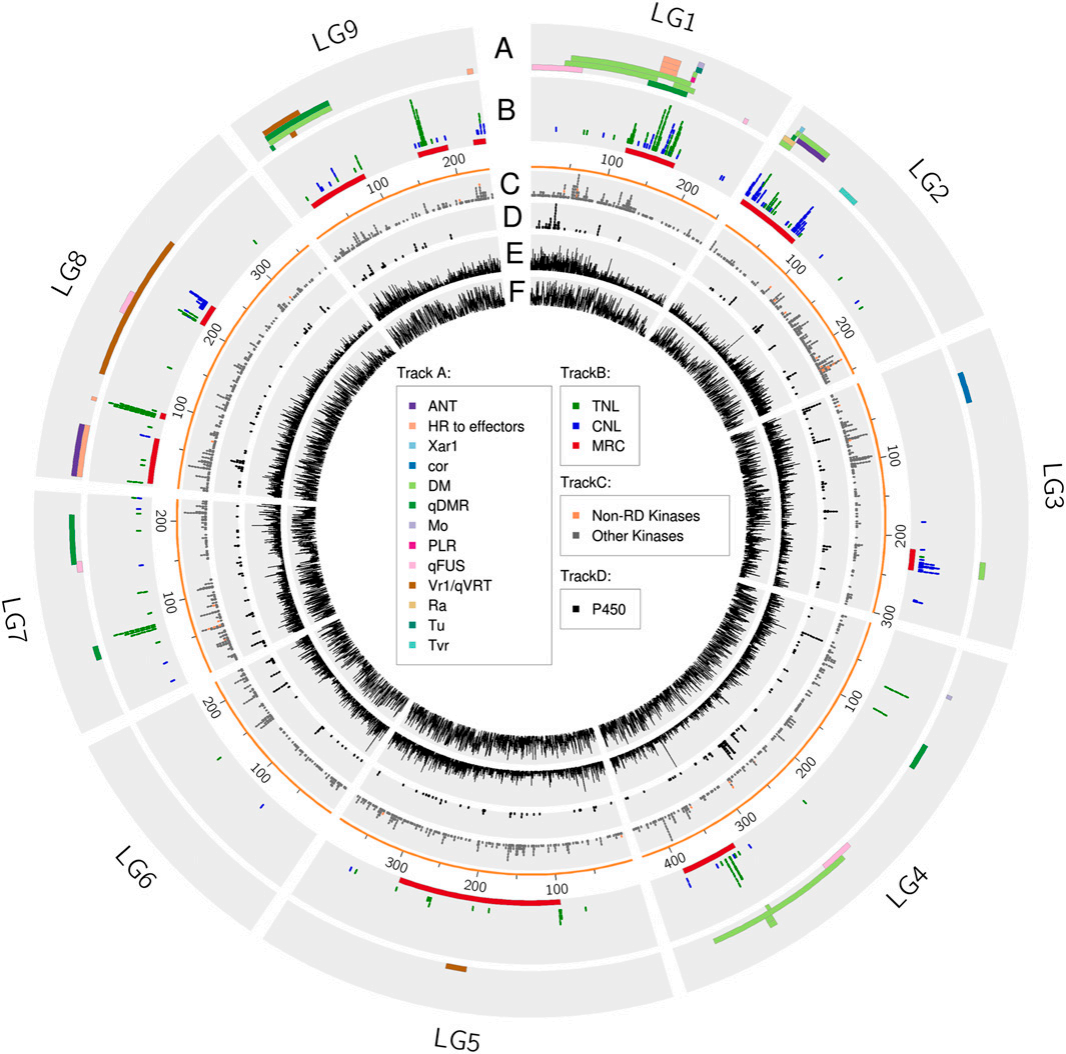
Genomic distribution of candidate genes involved in disease resistance over the nine chromosomes of lettuce (LG1−LG9; assembly version Lsat_1_v6_lg). Precise coordinates and references are provided in Table S2. Track A: Disease resistance phenotypes; bars reflect the resolution determined by mapping in populations other than the core reference mapping population. Track B: NLR-encoding genes; TNLs are colored green and CNLs blue. Each pixel represents a gene. MRCs are shown as red bars below the genes. The scale bar enumerates each chromosome in Mb. Track C: Genes encoding receptor-like kinases: non - arginine-aspartate (non-RD) RLKs are colored orange. Track D: Genes encoding cytochrome P450 proteins. Track E: Gene density. Track F: Repeat density.

A total of 703 LRR-encoding gene models were identified that did not contain a NB, CC, or TIR domain; 425 of these also had a predicted TM domain (Table 3). A protein kinase domain was detected in 298 of these 703 LRR-containing genes, 270 of which also had a TM domain and were therefore classified as putative RLKs. Clusters of RLK encoding genes are located on multiple chromosomes (Figure 2). These clusters are distinct from the clusters of NLR-encoding genes. Thirty-nine RLKs were non-RD kinases, 14 of which clustered within 22 Mb of each other at the opposite end of chromosome 2 to MRC2 (Table S2); no resistance phenotypes are known to map to this RLK cluster. Three RLPs with a predicted TM domain but no kinase domain and high sequence similarity to the tomato *Ve* gene are located within a 100 kb region of chromosome 9 that cosegregates with resistance to race 1 of *Verticillium dahliae* (Hayes *et al.* 2011).

### Grouping of NLR-encoding genes into 42 multigene families and singletons based on sequence similarity within the NB domain

**Figure 3:**
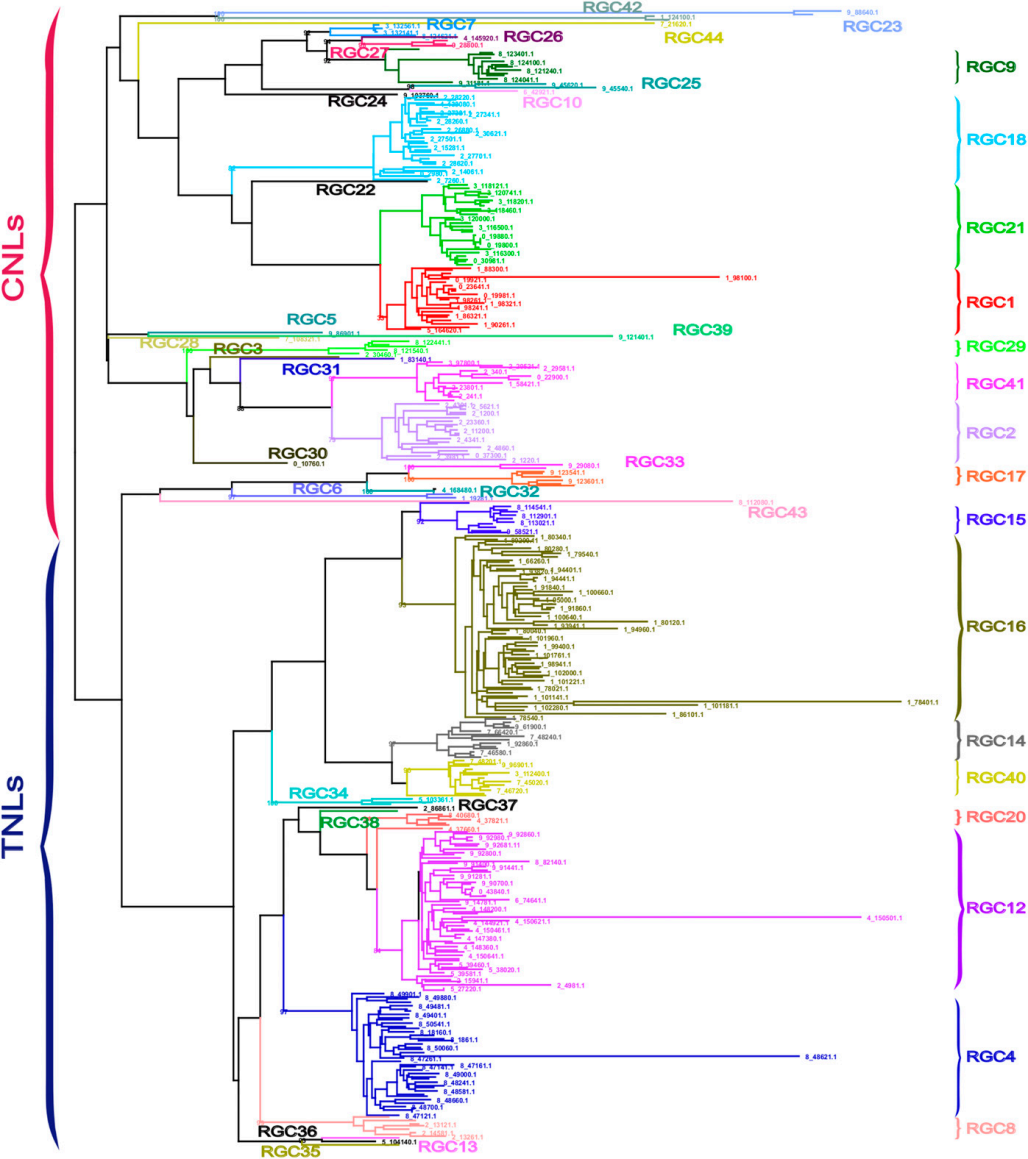
Dendrogram of lettuce nucleotide binding-leucine rich repeat receptors generated with RaxML. Bootstrap support values on major clades generated by 1000 repetitions. Nomenclature was kept consistent with previously described families (Meyers *et al.* 1998a; McHale *et al.* 2009).

A maximum-likelihood approach was used to study the sequence relationships of the NLR-encoding genes. Three of the 385 sequences were considered as a single sequence for alignment purposes because they were identical at the amino acid level in the NB region although they differed in their other domains. Clades with at least 70% sequence identity in pairwise comparisons of the NB domain were designated as distinct multigene families. This grouped the 385 NLR-encoding genes into 25 multigene families and 17 singletons (Figure 3). Nomenclature for each family was kept consistent with previous assignations as much as possible (Meyers *et al.* 1998b; McHale *et al.* 2009). Genes lacking a CC domain or TIR domain were designated either as CNL-like or TNL-like based on sequence similarity to the NB domains of CC-NB-LRR- and TIR-NB-LRR-encoding genes respectively.

A larger number of CNL than TNL families and singletons were identified (28 and 14 respectively), although fewer CNL-encoding genes than TNL-encoding genes were identified in total (167 and 21, respectively). Seventeen of the 42 clades were singletons, whereas 12 families contained more than 10 members each and accounted for 84% of the 385 NLR-encoding genes analyzed (Table 4). Three TNL families, *RGC4*, *RGC12*, and *RGC16*, were greatly expanded with more than 40 members each, comprising 73% of all the TNLs in the lettuce reference genome. The largest family was *RGC16* with 62 members.

**Table 4:** Number of NLR encoding genes per family and type

Singletons	NLR type	Multigene Family	Number of Genes	NLR type
*RGC3*	CNL	*RGC18*	29	CNL
*RGC5*	CNL	*RGC21*	28	CNL
*RGC10*	CNL	*RGC1*	22	CNL
*RGC22*	CNL	*RGC2*	20	CNL
*RGC24*	CNL	*RGC41*	14	CNL
*RGC26*	CNL	*RGC9*	12	CNL
*RGC28*	CNL	*RGC17*	6	CNL
*RGC30*	CNL	*RGC29*	5	CNL
*RGC31*	CNL	*RGC7*	4	CNL
*RGC39*	CNL	*RGC27*	3	CNL
*RGC43*	CNL	*RGC6*	2	CNL
*RGC44*	CNL	*RGC23*	2	CNL
*RGC13*	TNL	*RGC25*	2	CNL
*RGC35*	TNL	*RGC32*	2	CNL
*RGC36*	TNL	*RGC33*	2	CNL
*RGC37*	TNL	*RGC42*	2	CNL
*RGC38*	TNL	*RGC16*	62	TNL
		*RGC12*	55	TNL
		*RGC4*	42	TNL
		*RGC14*	14	TNL
		*RGC40*	13	TNL
		*RGC15*	11	TNL
		*RGC8*	7	TNL
		*RGC20*	6	TNL
		*RGC34*	3	TNL

Singletons have only a single member. NLR, nucleotide binding-leucine rich repeat receptor.

Draft genome assemblies of lettuce cvs. Diana, Valmaine, Greenlake, La Brillante, and PI125246 of *L. sativa* and accession US96UC23 of the wild progenitor *L. serriola* were mined for the singletons identified in cv. Salinas. Single homologs with 99–100% sequence identity for the entire length of the coding sequence at the nucleotide level were identified for all singleton *RGC*s in all six additional genotypes. Therefore, the *RGC* singletons were highly conserved and did not exhibit presence or absence polymorphism across this diverse germplasm. The complex sequence relationships of the NLR-encoding multigene families make assembly and identification of orthologs difficult; this precluded their facile analysis across these draft assemblies.

### Distribution of NLR-encoding genes and MRCs in the genome of lettuce

NLR-encoding genes are found in every chromosome. Chromosomes 5 and 6 have the lowest numbers, with only 13 and 2 such genes, respectively (Figure 2; Table S2). Chromosomes 2 and 3 predominantly contain CNLs (≥50% of all CNLs), whereas chromosomes 1, 4, 5, 7, 8, and 9 contain primarily TNLs (≥89% of all TNLs). Members of each multigene family are typically found in clusters on the same chromosome (at least 68% of the gene families) with the exception of *RGC12* that has a genome-wide distribution and *RGC14* that is present on four chromosomes. *RGC12* is the second largest family with 55 members present on six of the nine chromosomes; 20 members are present on chromosome 9, followed by 19 on chromosome 4, six on chromosomes 2 and 5, and one on chromosomes 6 and 8 (two are unmapped). Eight *RGC14* genes are on chromosome 7 with one, two, and three, on chromosomes 1, 5, and 9 respectively. The genome distribution of singletons was varied; *RGC37* was located with other NLR-encoding genes; however, the remainder was several Mb away from other NLRs (*RGC30* has not been mapped).

**Figure 4:**
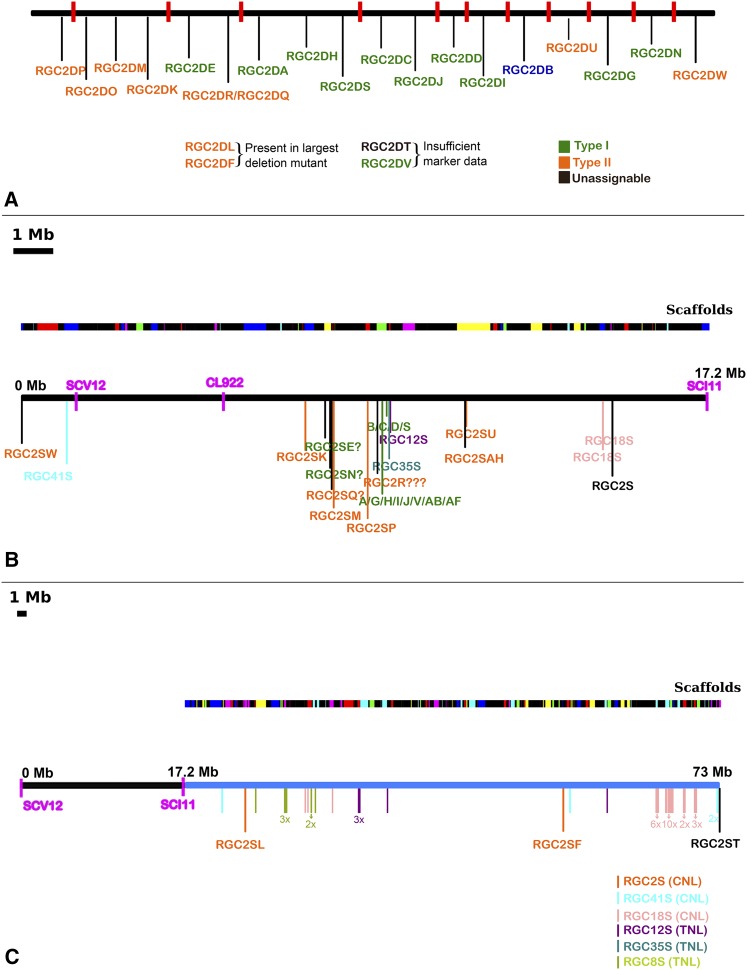
Comparison of the MRC2 locus in cvs. Diana (A) and Salinas (B and C). (A) Summary of the data previously published for the region as part of the map-based cloning of *Dm3* (Meyers *et al.* 1998a). The order of 24 *RGC2* members in cv. Diana, sequenced from 22 BACs, was based on the deletion breakpoints (shown as red bars) of nine fast neutron–induced *dm3* mutants. Genes depicted in green are Type I and in orange Type II genes (Kuang *et al.* 2004). *RGC2DB* is *Dm3* and it is a Type I gene. The spacing shown is for illustrative clarity; the physical distance between the BACs is unknown. (B) The *Dm3* mediated resistance was originally mapped to a region defined by markers SCV12 and SCI11 (Chin *et al.* 2001). This region spans approximately 17 Mb in cv. Salinas. The positions of the nucleotide binding-leucine rich repeat receptor (NLR) encoding genes are shown on the lower bar and they are color-coded with a different color for each gene family. NLR genes that do not belong to the *RGC2* gene family are colored light pink (*RGC18*) and light blue (*RGC41*). The upper bar shows the distribution of scaffolds for this piece of chromosome. Black represents scaffolds without any predicted genes. The rest of the scaffolds are colored randomly. (C) The distribution of NLRs across the entire MRC2 locus, including the *Dm3* region, is shown to scale on the lower bar. The scaffolds are shown on the upper bar.

**Figure 5:**
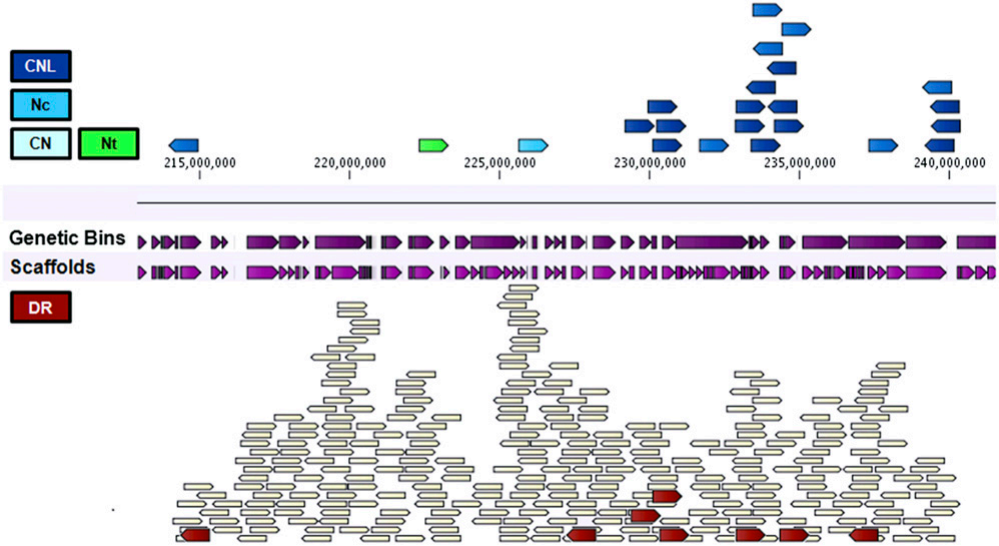
Graphical representation of the major resistance cluster on chromosome 3 (MRC3) of the reference genome assembly of *L. sativa* cv. Salinas. The top panel shows the position of nucleotide binding-leucine rich repeat receptor (NLR) encoding genes relative to the genetic bins and the scaffolds, whereas the bottom panel shows the position of all other genes that map to this locus. Color-coding: TNLs are colored in shades of green and CNLs in shades of blue, defense response (DR) genes in red and the remainder of the genes in yellow. The NLRs are further subdivided based on the number of characteristic domains detected. Abbreviations used: N for NB, L for leucine-rich repeat, C for coiled-coil, T for TOLL/interleukin-1 receptor. Lowercase letters used whenever the domain itself was not detected but inferred based on the phylogeny of the NB domain.

NLR-encoding genes are concentrated in five MRCs as defined by multiple disease resistance phenotypes on chromosomes 1, 2, 3, 4, and 8 (Table S2; Figure 2) (McHale *et al.* 2009; Truco *et al.* 2013). The MRCs contain both NLR-encoding genes as well as genes not related to disease resistance. They are in large gene-poor, transposon-rich regions of the cv. Salinas genome. The average gene density for the entire genome is 2.43 genes per 100 kb; however, the gene density within each MRC ranges from 1 to 2.08 and is even lower for the flanking region extending 50 Mb on either end of each MRC (Table 5). MRC1 and MRC4 both span ∼5 Mb and are composed of multiple families of NLR-encoding genes as described earlier (Christopoulou *et al.* 2015). MRC2 extends over ∼73 Mb and cosegregates with the large cluster of *Dm* genes that map to chromosome 2 (Figure 4). MRC3 spans approximately 25 Mb near the telomere of chromosome 3, contains almost exclusively *RGC21* genes (Figure 5), and cosegregates with *Dm13*. Two clusters of NLRs on chromosome 8, MRC8A (Figure S3) and MRC8B (Figure S4), are defined by the disease resistance phenotypes that map to chromosome 8. MRC8A spans ∼52 Mb; MRC8B is smaller and spans only 7 Mb. A third cluster of *RGCs* spans almost 26 Mb at the end of chromosome 8 and cosegregates with two QTL for resistance to *Fusarium* and *Verticillium* wilts respectively (Figure S5). Three distinct clusters of NLR encoding genes, MRC9A (Figure S6), MRC9B, and MRC9C, are present on chromosome 9; each spans approximately 72 Mb, 35 Mb, and 15 Mb. MRC9A cosegregates with two QTL for resistance to DM and the candidate RLK–encoding genes for resistance to *Verticillium dahliae*. MRC9B consists of a large cluster of 19 *RGC12*s; however, no known resistance phenotypes have so far been mapped to this locus.

**Table 5:** Sizes and gene densities of the MRC

MRC	Number of NLRs	Number of Non-NLRs	MRC Size, Mb	Total Gene Density (Genes/100 kb)	Gene Density Of Flanking Regions (Genes/100 kb)	
					50 Mb before	50 Mb after
MRC1	78	788	66	1.30	2.09	0.75
MRC2	61	691	73	1.03	1.82*^a^*	0.97
MRC3	22	300	25	1.27	0.86	0.75
MRC4	21	627	63	1.02	0.82	1.26*^b^*
MRC8A	6	1126	52	2.08	1.69*^c^*	1.44
MRC8B	36	93	7	1.74	1.68	1.71
MRC8C	26	322	26	1.34	1.28	0.90
MRC9A	11	1454	72	2.06	2.55*^d^*	0.88

The genome-wide average is 2.43 genes/100 kb. MRC, major resistance cluster; NLR, nucleotide binding-leucine rich repeat receptor.

a This region is only 1.76 Mb, because MRC2 starts almost at the beginning of the chromosome.

b This region is only 41 Mb, because MRC4 is at the end of the chromosome.

c This region is only 3.19 Mb, because MRC8A starts almost at the beginning of the chromosome.

d This region is only 19.6 Mb, because MRC9A starts almost at the beginning of the chromosome.

### Partial conservation of MRC2 structures in cvs. Salinas and Diana

Eight *Dm* genes and resistance to root aphid (*Ra*) are clustered near one end of chromosome 2 that genetically define the borders of MRC2 (McHale *et al.* 2009) (Table S2). The structure of MRC2 had been previously characterized extensively in cv. Diana as part of the map-based cloning of *Dm3* (Meyers *et al.* 1998a; Shen *et al.* 2002). A total of 23 BAC clones from cv. Diana were isolated containing 24 *RGC2* family members, 16 of which were ordered using the breakpoints in a series of deletion mutants (Meyers *et al.* 1998a). Also, seven additional *RGC2* members were partially sequenced from multiple genotypes to study the evolution of this cluster (Kuang *et al.* 2004).

We revisited the structure of the MRC2 cluster taking into consideration the genome assembly of cv. Salinas. The total size of MRC2 in cv. Salinas is ∼73 Mb and contains 754 genes of which 61 are NLRs, 2 are RLKs, and 5 are putative RLPs encoding genes (Figure 4). *Dm3* was originally mapped in cv. Diana to a region that spans approximately 17 Mb within MRC2 in cv. Salinas. Many rearrangements of the *RGC2* genes are evident between the two haplotypes (Figure 4). Fourteen of 21 *RGC2* genes present in Diana could be identified in the MRC2 assembly of cv. Salinas; 12 of these had been previously classified as conserved Type II genes in cv. Diana. Variable Type I genes, which are primarily found in the middle section of MRC2 in cv. Diana, are largely absent in cv. Salinas, possibly due to a large deletion. Only *RGC2SE* and *RGC2SN* Type I genes in cv. Diana had reciprocal best hits with single genes in cv. Salinas. There are two additional NLR genes in cv. Salinas that had high levels of sequence identity to multiple Type I genes from cv. Diana and therefore orthologous relationships could not be established. These results support the heterogeneous evolution of NLR genes at this locus with genes at the periphery evolving more slowly than genes in the middle of the cluster.

### Differential silencing of NLRs encoded at MRC2 by NB and LRR trigger sequences

The role of *RGC2* gene family members in determining resistance specificities cosegregating with *Dm3* had been studied previously using RNAi (Wroblewski *et al.* 2007). Not only *Dm3*, but also *Dm14*, *Dm16* and *Dm18*, as well as *Ra* were abrogated when an RNAi construct targeting the LRR-encoding domain of *RGC2B* was tested in genotypes expressing each of these resistance phenotypes. Sequence identities typically differ between NB and LRR domains, with NB domains tending to be more conserved than LRR domains. Therefore, a fragment of the NB domain of *RGC2B* was used as the trigger sequence for RNAi to determine whether more, the same, or a different spectrum of *Dm* specificities were abrogated. Transgenics were generated in cv. Diana and T_1_ lines were evaluated for RNAi efficacy. The Diana T_2_s tested for *Dm3*-mediated resistance segregated for the resistant phenotype as expected from hemizygous T_1_s. Presence of the transgene, confirmed by both PCR and GUS expression assays, was absolutely coincident with loss of resistance (Figure 1).

The two cv. Diana transgenics with the greatest levels of silencing were crossed to different genotypes to test for *Dm2*, *Dm6*, *Dm15*, *Dm16*, and *Dm18* specificities. All the F_1_s tested segregated for the presence of the RNAi construct. *Dm3* and *Dm18* were abrogated by trigger sequences derived from both the NB and LRR domains of *Dm3*; however, *Dm14* and *Dm16* were abrogated by the LRR-derived trigger sequence but not the NB-derived trigger sequence (*Dm14* was not tested with the NB-RNAi construct). Conversely, *Dm6* was compromised by the NB-derived trigger sequences but not those derived from the LRR. This was surprising because NB domains tend to be more conserved than LRR domains; this suggests that *Dm16* is a chimeric Type I gene with a LRR domain that is more closely related to *Dm3* than *Dm6* but with a more distantly related NB domain.

### Structure and functional analysis of NLRs encoded at MRC3 in cv. Salinas

MRC3 is a tight cluster consisting primarily of *RGC21* family members on chromosome 3 that cosegregates with *Dm13*. It spans ∼25 Mb and contains 322 genes of which 31 are predicted to be involved in pathogen recognition: 27 CNL (26 *RGC21*, 1 *RGC40*), two RLK, and two LRR encoding genes (Figure 5; Table 5).

Transgenics of cv. Cobham Green were generated for two constructs that targeted members of the *RGC21* family of NLR-encoding genes that mapped to chromosome 3. These trigger sequences were derived from ESTs that were subsequently shown to encode the NB and LRR domains of different *RGC21* family members (Table 1). Two T_1_ transgenic lines per construct were silenced for *GUS* and therefore presumably silenced for the targeted NLR-encoding gene(s). F_1_s of crosses to introduce the transgenes into cv. Pennlake carrying *Dm13* segregated for the transgene as evidenced by both assays of transient expression of *GUS* and PCR amplification of the transgene. The F_1_s of Pennlake x QGC7A16_LRR_RNAi also segregated 1:1 for susceptibility:resistance to isolate CS12 of *B. lactucae*. The loss of resistance cosegregated perfectly with the presence of the transgene for F_1_s derived from two independent primary transgenics (Figure 1). Therefore, *QGC7A16* or another *RGC21* family member(s) confers *Dm13*. However, LserNBS02_NB_RNAi that also targeted *RGC21* family members did not abrogate *Dm13*-mediated resistance, even though F_1_s carrying the transgene were effectively silenced for GUS expression.

QGC7A16_LRR_RNAi was predicted as potentially targeting 16 of 28 *RGC21* family members and eight other genes encoding only an LRR domain, whereas LserNBS02_NB_RNAi was predicted to target 26 of 33 members of the *RGC21* family, 3 *RGC1* members and 3 unassigned CNLs. All of NLR-encoding genes predicted to be silenced by QGC7A16_LRR_RNAi also were predicted to be silenced by LserNBS02_NB_RNAi (Table S5). The lack of abrogation of resistance by LserNBS02_NB_RNAi despite it being predicted to target most known *RGC21* members could be explained by additional diverse *RGC21* genes unique to cv. Pennlake that are not present in the cv. Salinas assembly; however, both Salinas and Pennlake exhibit *Dm13* specificity. In addition to silencing NLR-encoding genes, QGC7A16_LRR_RNAi was predicted to silence eight LRR encoding genes that lack an NB domain. Seven out of the eight LRR encoding genes only targeted by QGC7A16_LRR_RNAi have high sequence similarity to other *RGC21* members. These could either be *RGC21* genes that are improperly assembled in the reference assembly of cv. Salinas or correctly assembled genes that lack an NB domain, one or more of which can confer *Dm13* specificity. Alternatively, *Dm13* may be encoded by a full length *RGC21* gene and inadequate levels of silencing occurred from LserNBS02_NB_RNAi.

### Structure and functional analysis of NLRs encoded at MRC8

There are three MRCs on chromosome 8 that cosegregate with different resistance-related phenotypes. *Agrobacterium*-mediated transient expression of bacterial effectors AvrRps4 and AvrPpiC triggers a HR in several cultivars. MRC8A is a small cluster spread over a 54-Mb region on chromosome 8 in cv. Salinas that cosegregates with HR to AvrPpiC in cv. Valmaine. MRC8A has a total of 1132 genes, of which 23 genes are potentially involved in pathogen recognition: four TNLs (*RGC4*) and two CNLs (*RGC27*), eight RLKs, four RLPs, one TIR-, and four LRR-encoding genes (Figure S3; Table 5). Three constructs were tested for abrogation of HR to AvrPpiC: Lsat11_NB_RNAi, Contig5632_TIR_RNAi, and QGD14014_NB_RNAi (Table 1). These were predicted to potentially silence members of *RGC34*, *RGC4*, and *RGC5*, respectively (Table S3). Two RNAi transgenic lines exhibiting silencing per construct were crossed to cv. Valmaine and the hybrids were tested for silencing and the response to the AvrPpiC. All the tested F_1_ progenies segregated for high levels of silencing of GUS coincident with the presence of the transgene. A strong HR occurred in both the presence and the absence of the transgene. Therefore, there is no evidence that any of these NLRs determine the HR to AvrPpiC.

MRC8B is a dense cluster cosegregating with the HR to AvrRps4. It spans ∼7 Mb in cv. Salinas and contains 127 genes, 36 of which are *RGC4* members (Figure S4; Table 5). Four constructs were tested: LEO266_TIR_RNAi (*RGC4*), LEO414_LRR_RNAi (*RGC9*), AY153833.1_LRR_RNAi (*RGC15*), and LEO_395_LRR_RNAi (*RGC29*) (Table 1). The last three constructs were tested because at the time of the experiments genetic data suggested that all of these RGCs co-segregated with HR to AvrRps4; however, they were subsequently shown not to be at this location in the whole genome assembly. Two independent transgenic lines for each construct were crossed to cv. Valmaine. The leaves of F_1_s were pressured infiltrated with *A. tumefaciens* cultures expressing *AvrRps4*. The F_1_s segregated for the transgene as shown by PCR and GUS transient assays; however, all of them retained a strong HR to AvrRps4 regardless of the silencing and the presence of the transgene (Table S4). Therefore, there is no evidence that members of *RGC4*, *RGC9*, or *RGC15*, *RGC29* targeted by these constructs have a role in the recognition of AvrRps4 in lettuce. *In silico* predictions of RNAi targets for these constructs showed that there are 3 and 30 NLR-encoding genes within MRC8A and MRC8B respectively that were unlikely to be silenced by these constructs (Table S3; Table S4). These are candidates for additional RNAi targets to test their involvement in HR to AvrPpiC and AvrRps4.

MRC8C is a complex cluster that spans 25 Mb and contains 348 genes, 26 of which are NLR-encoding genes (Table 5). There are 9 *RGC15*, 11 *RGC9*, 4 *RGC29*, 1 *RGC7*, and 1 *RGC43* members (Table S2; Figure S5). A QTL to *Fusarium oxysporum* and a minor QTL for resistance to *V. dahliae* cosegregate with MRC8C. These resistance phenotypes have to be assessed in the field and therefore no constructs were tested for these phenotypes.

## Discussion

This paper provides a detailed description of the genome-wide architecture of disease resistance in lettuce. Thirty-six major phenotypic genes and 20 QTLs for resistance to 10 pathogens and one pest have been positioned relative to 1134 candidate genes in genomic scaffolds assigned to genetic bins ordered along the nine chromosomal linkage groups (Table S2). Most but not all resistance phenotypes are located with arrays of NLR-encoding genes. Conversely, of the 42 families of NLR-encoding genes in *L. sativa* cv. Salinas, 26 were co-located with resistance phenotypes. We therefore defined five MRCs as regions of the genome enriched for determinants of resistance based on both phenotypic information from a variety of genotypes and the genomic distribution of NLR-encoding genes. This clustered distribution is similar to that observed in other species (Zhang *et al.* 2014). The total number of NLR-encoding genes observed in cv. Salinas (385) is in the middle of the range (54−1015) observed in other plant species (Meyers *et al.* 2003; Zhou *et al.* 2004; Kohler *et al.* 2008; Porter *et al.* 2009; Arya *et al.* 2014; Shao *et al.* 2014). We detected more TNL than CNL genes in total but fewer TNL than CNL families; this is due the expansion of three TNL families, *RGC4*, *RGC12*, and *RGC16*, that make up 68% of all NLR-encoding genes. Seventeen families were only represented by singletons, of which 12 were CNLs and 5 were TNLs (Table 4).

Most families tend to be colocated in the same MRC, likely reflecting expansion by unequal crossing over (Chin *et al.* 2001; Meyers *et al.* 2003; Zhou *et al.* 2004). The largest family, *RGC16*, has 62 members, of which 60 are at MRC1 (Table S2). However, there are exceptions to the colocation of family members. *RGC14* is distributed in two clusters (on chromosomes 7 and 9), possibly as a consequence of a segmental duplication event. The second largest family, *RGC12*, with 55 members is widely distributed through the genome and present on six chromosomes. The multiple chromosomal locations of large families may reflect grouping of more slowly evolving genes into single families based on our 70% threshold for sequence identity. Several clusters are homogeneous, comprising of a single *RGC* family (MRC1, MRC4, MRC3, MRC8A, MRC8B). Some are complex comprising of multiple TNLs and CNLs; the most complex was MRC2 that had representatives of three TNL and three CNL families (Table S2). All MRCs contained genes other than NLR-encoding genes; however, they were not enriched for any classes of genes, other than NLR-encoding genes.

The majority of the data presented are based on the reference genome of cv. Salinas. However, NLR-encoding gene repertoires can differ greatly between genotypes; the number of *RGC2* family members can vary from ∼12 to 42 (Kuang *et al.* 2006). The numbers detected in the genome assemblies of cvs. Salinas and Diana were in the middle of this range (20 and 29, respectively). Although all of the conserved Type II genes were present in both, cv. Diana had more of the variable Type I genes; however, the order of the Type II genes apparently differs between the haplotypes. Therefore, while the general architecture observed in cv. Salinas may be representative of *L. sativa*, the fine-scale details may be specific to individual genotypes; this needs to be taken into consideration when implementing functional studies and cloning strategies.

Functional studies using RNAi successfully assigned 16 resistance phenotypes to four *RGC* families. The selection of RGCs targeted for RNAi predated the sequencing and assembly of the reference genome and was based on ESTs of NLR-encoding genes that had been mapped relative to disease resistance phenotypes (McHale *et al.* 2009). This provided RNAi transgenics targeting 12 of the 42 RGC families representing most of the major gene families and the majority of NLRs that cosegregate with resistance phenotypes. Members of *RGC1* are required for the *Dm5/8*- and *Dm45*-mediated resistance as well as for the HR to AvrB, AvrRpm1, and AvrRpt2, that map to MRC1. *RGC12* family members are required for *Dm4*, *Dm7*, *Dm11*, and *Dm44* specificities in MRC4 as described previously (Christopoulou *et al.* 2015). For MRC2 and MRC4, members of the largest *RGC* family present were shown to be required for the co-segregating resistance specificities. Interestingly, six constructs predicted to target all but four members of the largest family at MRC1, *RGC16*, failed to abrogate any of the nine tested phenotypes that map to this cluster.

RNAi was an efficient method to assign resistance phenotypes to *RGC* families. Fragments of the NB, LRR and TIR domains were used as trigger sequences for RNAi for the 33 constructs used in this study. Abrogation of resistance was observed with all three types of trigger sequences. The different regions appeared to confer RNAi with similar efficacy; 1 of 4 TIR, 2 of 17 NB, and 4 of 12 LRR trigger sequences abrogated resistance. Although the NB domain is the most conserved NLR domain, targeting of the TIR and the LRR domains also resulted in silencing of multiple family members. When multiple trigger sequences targeted the same gene, as for *Dm3*, overlapping sets of specificities were abrogated. This provides the opportunity for designing trigger sequences that can abrogate specific subsets of family members.

These 33 transgenic RNAi lines are resources for assigning additional resistance phenotypes to RGC families. Also, further lines can be generated to target more of the *RGC* families within MRCs with resistance phenotypes for which causal *RGCs* have not been identified, such as MRC1. After the sequence analysis and the ultradense map were refined it is evident that some existing RNAi constructs could also be tested for other specificities that cosegregate with RGCs of the targeted family. For example constructs Contig5632_TIR_RNAi and AF017754_NB_RNAi were tested for phenotypes that map to MRC8A and MRC3 respectively but target a large subset of *RGC4* members and would be good tester stocks for the HR to AvrRps4 (MRC8B). The RNAi strategy that we have implemented provides an efficient approach to attribute phenotypes to subsets of a multigene *RGC* family; however, achievement of single gene resolution is challenging by RNAi methods. Nonetheless, it does provide candidates for knockouts of individual genes using CRISPR/Cas9 technology and valuable information for the development of robust markers to facilitate breeding efforts.

Overall, resistance phenotypes were shown to be encoded by both CNL and TNL multigene families. No resistance phenotype was encoded by a singleton such as the allelic series observed at the *L* locus in flax (Ellis *et al.* 2007). Interestingly, when a *Dm* specificity was abrogated, other resistance phenotypes that mapped to the same locus were also compromised indicating that the same *RGC* family encoded multiple *Dm* genes. Conversely, in all cases in which silencing of a *Dm* specificity occurred, the causal *RGC* family contained more than 20 members. Together, these data suggest that the expansion of specific *RGC* families in lettuce is in response to evolutionary pressures exerted by variation in *B. lactucae*.

## Supplementary Material

Supporting Information

## References

[bib1] Altschul S. F. Madden T. L. Schäffer A. A. Zhang J. Zhang Z., 1997 Gapped BLAST and PSI-BLAST: a new generation of protein database search programs. Nucleic Acids Res. 25: 3389–3402. 9254694 10.1093/nar/25.17.3389 PMC146917

[bib2] Arya P. Kumar G. Acharya V. Singh A. K., 2014 Genome-wide identification and expression analysis of NBS-encoding genes in *Malus domestica* and expansion of NBS genes family in Rosaceae. PLoS One 9: e107987. 25232838 10.1371/journal.pone.0107987 PMC4169499

[bib3] Bernatzky R. Tanksley S. D., 1986 Toward a saturated linkage map in tomato based on isozymes and random cDNA sequences. Genetics 112: 887–898. 17246322 10.1093/genetics/112.4.887 PMC1202783

[bib4] Bernsel A. Viklund H. Falk J. Lindahl E. von Heijne G., 2008 Prediction of membrane-protein topology from first principles. Proc. Natl. Acad. Sci. USA 105: 7177–7181. 18477697 10.1073/pnas.0711151105 PMC2438223

[bib5] Bozkurt T. O. Schornack S. Banfield M. J. Kamoun S., 2012 Oomycetes, effectors, and all that jazz. Curr. Opin. Plant Biol. 15: 483–492. 22483402 10.1016/j.pbi.2012.03.008

[bib6] Chin D. B. Arroyo-Garcia R. Ochoa O. E. Kesseli R. V. Lavelle D. O., 2001 Recombination and spontaneous mutation at the major cluster of resistance genes in Lettuce (*Lactuca sativa*). Genetics 157: 831–849. 11157000 10.1093/genetics/157.2.831 PMC1461523

[bib7] Christopoulou M. McHale L. Kozik A. Reyes-Chin Wo S. Wroblewski T., 2015 Dissection of two complex clusters of resistance genes in Lettuce (*Lactuca sativa*). Mol. Plant Microbe Interact. 28: 751−765. 25650829 10.1094/MPMI-06-14-0175-R

[bib8] Clark R. M. Schweikert G. Toomajian C. Ossowski S. Zeller G., 2007 Common sequence polymorphisms shaping genetic diversity in *Arabidopsis thaliana*. Science 317: 338–342. 17641193 10.1126/science.1138632

[bib9] Crute I. Johnson A., 1976 The genetic relationship between races of *Bremia lactucae* and cultivars of *Lactuca sativa*. Ann. Appl. Biol. 83: 125–137.

[bib10] Dardick C. Ronald P., 2006 Plant and animal pathogen recognition receptors signal through non-RD kinases. PLoS Pathog. 2: 14–28. 10.1371/journal.ppat.0020002 PMC1331981 16424920

[bib11] Dardick C. Schwessinger B. Ronald P., 2012 Non-arginine-aspartate (non-RD) kinases are associated with innate immune receptors that recognize conserved microbial signatures. Curr. Opin. Plant Biol. 15: 358–366. 22658367 10.1016/j.pbi.2012.05.002

[bib12] Darriba D. Taboada G. L. Doallo R. Posada D., 2011 ProtTest 3: fast selection of best-fit models of protein evolution. Bioinformatics 27: 1164–1165. 21335321 10.1093/bioinformatics/btr088 PMC5215816

[bib13] Davis R. M. Subbarao K. V. Raid R. N. Kurtz E. A., 1997 *Compendium of Lettuce Diseases*. APS Press, St. Paul, Minnesota.

[bib14] Dower W. J. Miller J. F. Ragsdale C. W., 1988 High efficiency transformation of *E.coli* by high voltage electroporation. Nucleic Acids Res. 16: 6127–6145. 3041370 10.1093/nar/16.13.6127 PMC336852

[bib15] Ellis J. G. Dodds P. N. Lawrence G. J., 2007 Flax rust resistance gene specificity is based on direct resistance-avirulence protein interactions. Annu. Rev. Phytopathol. 45: 289–306. 17430087 10.1146/annurev.phyto.45.062806.094331

[bib16] Ellis P. McClement S. Saw P. Phelps K. Vice W., 2002 Identification of sources of resistance in lettuce to the lettuce root aphid, *Pemphigus bursarius*. Euphytica 125: 305–315.

[bib17] Farrara B. F. Ilott T. W. Michelmore R. W., 1987 Genetic analysis of factors for resistance to downy mildew (*Bremia lactucae*) in species of lettuce (*Lactuca sativa* and *L. serriola*). Plant Pathol. 36: 499–514.

[bib18] Guo Y. L. Fitz J. Schneeberger K. Ossowski S. Cao J., 2011 Genome-wide comparison of nucleotide-binding site-leucine-rich repeat-encoding genes in *Arabidopsis*. Plant Physiol. 157: 757–769. 21810963 10.1104/pp.111.181990 PMC3192553

[bib19] Hayes R. J. McHale L. K. Vallad G. E. Truco M. J. Michelmore R. W., 2011 The inheritance of resistance to Verticillium wilt caused by race 1 isolates of *Verticillium dahliae* in the lettuce cultivar La Brillante. Theor. Appl. Genet. 123: 509–517. 21567237 10.1007/s00122-011-1603-y

[bib65] Hoekema A. Hirsch P. R. Hooykaas P. J. J. Schilperoort R. A., 1983 A binary plant vector strategy based on separation of vir-region and T-region of the *Agrobacterium-tumefaciens* Ti-plasmid. Nature 303: 179–180.

[bib20] Hulbert S. H. Webb C. A. Smith S. M. Sun Q., 2001 Resistance gene complexes: evolution and utilization. Annu. Rev. Phytopathol. 39: 285–312. 11701867 10.1146/annurev.phyto.39.1.285

[bib21] Huson D. H. Richter D. C. Rausch C. Dezulian T. Franz M., 2007 Dendroscope: An interactive viewer for large phylogenetic trees. BMC Bioinformatics 8: 460. 18034891 10.1186/1471-2105-8-460 PMC2216043

[bib22] Ilott T. Hulbert S. Michelmore R., 1989 Genetic analysis of the gene-for-gene interaction between lettuce (*Lactuca sativa*) and *Bremia lactucae*. Phytopathology 79: 888–897.

[bib23] Jones J. D. Dangl J. L., 2006 The plant immune system. Nature 444: 323–329. 17108957 10.1038/nature05286

[bib24] Karasov T. L. Horton M. W. Bergelson J., 2014 Genomic variability as a driver of plant–pathogen coevolution? Curr. Opin. Plant Biol. 18: 24–30. 24491596 10.1016/j.pbi.2013.12.003 PMC4696489

[bib25] Katoh K. Standley D. M., 2013 MAFFT multiple sequence alignment software version 7: improvements in performance and usability. Mol. Biol. Evol. 30: 772–780. 23329690 10.1093/molbev/mst010 PMC3603318

[bib26] Kobe B. Deisenhofer J., 1994 The leucine-rich repeat: a versatile binding motif. Trends Biochem. Sci. 19: 415–421. 7817399 10.1016/0968-0004(94)90090-6

[bib27] Kohler A. Rinaldi C. Duplessis S. Baucher M. Geelen D., 2008 Genome-wide identification of NBS resistance genes in *Populus trichocarpa*. Plant Mol. Biol. 66: 619–636. 18247136 10.1007/s11103-008-9293-9

[bib28] Krogh A. Larsson B. Von Heijne G. Sonnhammer E. L., 2001 Predicting transmembrane protein topology with a hidden Markov model: application to complete genomes. J. Mol. Biol. 305: 567–580. 11152613 10.1006/jmbi.2000.4315

[bib29] Kuang H. Woo S.-S. Meyers B. C. Nevo E. Michelmore R. W., 2004 Multiple genetic processes result in heterogeneous rates of evolution within the major cluster disease resistance genes in lettuce. Plant Cell 16: 2870–2894. 15494555 10.1105/tpc.104.025502 PMC527186

[bib30] Kuang H. Ochoa O. E. Nevo E. Michelmore R. W., 2006 The disease resistance gene *Dm3* is infrequent in natural populations of *Lactuca serriola* due to deletions and frequent gene conversions at the *RGC2* locus. Plant J. 47: 38–48. 16762035 10.1111/j.1365-313X.2006.02755.x

[bib31] Leister D., 2004 Tandem and segmental gene duplication and recombination in the evolution of plant disease resistance genes. Trends Genet. 20: 116–122. 15049302 10.1016/j.tig.2004.01.007

[bib32] Luo S. Zhang Y. Hu Q. Chen J. Li K., 2012 Dynamic nucleotide-binding site and leucine-rich repeat-encoding genes in the grass family. Plant Physiol. 159: 197–210. 22422941 10.1104/pp.111.192062 PMC3375961

[bib33] McDonnell A. V. Jiang T. Keating A. E. Berger B., 2006 Paircoil2: improved prediction of coiled coils from sequence. Bioinformatics 22: 356–358. 16317077 10.1093/bioinformatics/bti797

[bib34] McHale L. Tan X. Koehl P. Michelmore R., 2006 Plant NBS-LRR proteins: adaptable guards. Genome Biol. 7: 212. 16677430 10.1186/gb-2006-7-4-212 PMC1557992

[bib35] McHale L. K. Truco M. J. Kozik A. Wroblewski T. Ochoa O. E., 2009 The genomic architecture of disease resistance in lettuce. Theor. Appl. Genet. 118: 565–580. 19005638 10.1007/s00122-008-0921-1

[bib36] Meyers B. C. Chin D. B. Shen K. A. Sivaramakrishnan S. Lavelle D. O., 1998 a The major resistance gene cluster in lettuce is highly duplicated and spans several megabases. Plant Cell 10: 1817–1832. 9811791 10.1105/tpc.10.11.1817 PMC143960

[bib37] Meyers B. C. Shen K. A. Rohani P. Gaut B. S. Michelmore R. W., 1998 b Receptor-like genes in the major resistance locus of lettuce are subject to divergent selection. Plant Cell 10: 1833–1846. 9811792 10.1105/tpc.10.11.1833 PMC143952

[bib38] Meyers B. C. Kozik A. Griego A. Kuang H. Michelmore R. W., 2003 Genome-wide analysis of NBS-LRR–encoding genes in Arabidopsis. Plant Cell 15: 809–834. 12671079 10.1105/tpc.009308 PMC152331

[bib39] Meyers B. C. Kaushik S. Nandety R. S., 2005 Evolving disease resistance genes. Curr. Opin. Plant Biol. 8: 129–134. 15752991 10.1016/j.pbi.2005.01.002

[bib40] Michelmore R. W. Meyers B. C., 1998 Clusters of resistance genes in plants evolve by divergent selection and a birth-and-death process. Genome Res. 8: 1113–1130. 9847076 10.1101/gr.8.11.1113

[bib41] Michelmore R. Wong J., 2008 Classical and molecular genetics of *Bremia lactucae*, cause of lettuce downy mildew. Eur. J. Plant Pathol. 122: 19–30.

[bib42] Michelmore R. Marsh E. Seely S. Landry B., 1987 Transformation of lettuce (*Lactuca sativa*) mediated by *Agrobacterium tumefaciens*. Plant Cell Rep. 6: 439–442. 24248927 10.1007/BF00272777

[bib43] Michelmore R. Ochoa O. Wong J., 2009 *Bremia lactucae* and lettuce downy mildew, *Oomycete Genetics and Genomics: Diversity*, *Plant and Animal Interactions*, *and Toolbox*, edited by Kamoun S. Lamour K. John Wiley, Hoboken, New Jersey.

[bib44] Miller, M. A., W. Pfeiffer, and T. Schwartz, 2010 Creating the CIPRES Science Gateway for inference of large phylogenetic trees, pp. 1–8 in *Gateway Computing Environments Workshop (GCE)*, 2010. New Orleans, LA, November 14, 2010 IEEE.

[bib45] Monaghan J. Zipfel C., 2012 Plant pattern recognition receptor complexes at the plasma membrane. Curr. Opin. Plant Biol. 15: 349–357. 22705024 10.1016/j.pbi.2012.05.006

[bib46] Mondragon-Palomino M. Gaut B. S., 2005 Gene conversion and the evolution of three leucine-rich repeat gene families in *Arabidopsis thaliana*. Mol. Biol. Evol. 22: 2444–2456. 16120808 10.1093/molbev/msi241

[bib47] Nicholas K. B. Nicholas H. Deerfield D., 1996 GeneDoc: analysis and visualization of genetic variation. Embnew.news 4: 14.

[bib48] Porter B. Paidi M. Ming R. Alam M. Nishijima W., 2009 Genome-wide analysis of *Carica papaya* reveals a small NBS resistance gene family. Mol. Genet. Genomics 281: 609–626. 19263082 10.1007/s00438-009-0434-x

[bib49] Richly E. Kurth J. Leister D., 2002 Mode of amplification and reorganization of resistance genes during recent *Arabidopsis thaliana* Evolution. Mol. Biol. Evol. 19: 76–84. 11752192 10.1093/oxfordjournals.molbev.a003984

[bib50] Ronald P. C. Beutler B., 2010 Plant and animal sensors of conserved microbial signatures. Science 330: 1061–1064. 21097929 10.1126/science.1189468

[bib51] Schöb H. Kunz C. Meins F. Jr, 1997 Silencing of transgenes introduced into leaves by agroinfiltration: a simple, rapid method for investigating sequence requirements for gene silencing. Molecular and General Genetics MGG 256: 581–585. 9413443 10.1007/s004380050604

[bib52] Shao Z.-Q. Zhang Y.-M. Hang Y.-Y. Xue J.-Y. Zhou G.-C., 2014 Long-term evolution of nucleotide-binding site-leucine-rich repeat genes: understanding gained from and beyond the legume family. Plant Physiol. 166: 217–234. 25052854 10.1104/pp.114.243626 PMC4149708

[bib53] Shen J. Araki H. Chen L. Chen J.-Q. Tian D., 2006 Unique evolutionary mechanism in R-genes under the presence/absence polymorphism in *Arabidopsis thaliana*. Genetics 172: 1243–1250. 16452149 10.1534/genetics.105.047290 PMC1456222

[bib54] Shen K. A. Chin D. B. Arroyo-Garcia R. Ochoa O. E. Lavelle D. O., 2002 *Dm3* is one member of a large constitutively expressed family of nucleotide binding site-leucine-rich repeat encoding genes. Mol. Plant Microbe Interact. 15: 251–261. 11952128 10.1094/MPMI.2002.15.3.251

[bib55] Stamatakis A., 2014 RAxML version 8: a tool for phylogenetic analysis and post-analysis of large phylogenies. Bioinformatics 30: 1312–1313. 24451623 10.1093/bioinformatics/btu033 PMC3998144

[bib56] Stamatakis A. Hoover P. Rougemont J., 2008 A rapid bootstrap algorithm for the RAxML web servers. Syst. Biol. 57: 758–771. 18853362 10.1080/10635150802429642

[bib57] Takken F. L. W. Goverse A., 2012 How to build a pathogen detector: structural basis of NB-LRR function. Curr. Opin. Plant Biol. 15: 375–384. 22658703 10.1016/j.pbi.2012.05.001

[bib58] Truco M. J. Ashrafi H. Kozik A. van Leeuwen H. Bowers J., 2013 An ultra high-density, transcript-based, genetic map of lettuce. G3 (Bethesda) 3: 617–631. 10.1534/g3.112.004929 PMC3618349 23550116

[bib59] Wroblewski T. Tomczak A. Michelmore R., 2005 Optimization of *Agrobacterium*-mediated transient assays of gene expression in lettuce, tomato and Arabidopsis. Plant Biotechnol. J. 3: 259–273. 17173625 10.1111/j.1467-7652.2005.00123.x

[bib60] Wroblewski T. Piskurewicz U. Tomczak A. Ochoa O. Michelmore R. W., 2007 Silencing of the major family of NBS-LRR-encoding genes in lettuce results in the loss of multiple resistance specificities. Plant J. 51: 803–818. 17587302 10.1111/j.1365-313X.2007.03182.x

[bib61] Wroblewski T. Matvienko M. Piskurewicz U. Xu H. Martineau B., 2014 Distinctive profiles of small RNA couple inverted repeat-induced post-transcriptional gene silencing with endogenous RNA silencing pathways in *Arabidopsis*. RNA 20: 1987–1999. 25344399 10.1261/rna.046532.114 PMC4238362

[bib62] Yue J. X. Meyers B. C. Chen J. Q. Tian D. C. Yang S. H., 2012 Tracing the origin and evolutionary history of plant nucleotide-binding site-leucine-rich repeat (NBS-LRR) genes. New Phytol. 193: 1049–1063. 22212278 10.1111/j.1469-8137.2011.04006.x

[bib63] Zhang R. Z. Murat F. Pont C. Langin T. Salse J., 2014 Paleo-evolutionary plasticity of plant disease resistance genes. BMC Genomics 15: 187. 24617999 10.1186/1471-2164-15-187 PMC4234491

[bib64] Zhou T. Wang Y. Chen J. Q. Araki H. Jing Z., 2004 Genome-wide identification of NBS genes in japonica rice reveals significant expansion of divergent non-TIR NBS-LRR genes. Mol. Genet. Genomics 271: 402–415. 15014983 10.1007/s00438-004-0990-z

