# Supplementary material for: Genome-Wide Architecture of Disease Resistance Genes in Lettuce
Source: G3 (Bethesda). 2015 Oct 8;5(12):2655–69. doi: 10.1534/g3.115.020818 (PMC4683639; doi:10.1534/g3.115.020818)
Supplement: Supporting Information [file supp_5_12_2655__index.html]

Genome-Wide Architecture of Disease Resistance Genes in Lettuce — Supporting Information 

# Genome-Wide Architecture of Disease Resistance Genes in Lettuce

## Supporting Information for Christopoulou *et al.*, 2015

**Files in this Data Supplement:**

- File S1 - Supporting Materials and Methods. (.docx, 16 KB)
- Figure S1 - Vectors used for RNAi constructs. (.docx, 69 KB)
- Figure S2 - Graphical representation of the major resistance cluster on chromosome 2 (MRC2) of the reference genome assembly of *L. sativa* cv. Salinas. (.docx, 113 KB)
- Figure S3 - Graphical representation of the first major resistance cluster on chromosome 8 (MRC8A) of the reference genome assembly of *L. sativa* cv. Salinas. (.docx, 404 KB)
- Figure S4 - Graphical representation of the second major resistance cluster on chromosome 8 (MRC8B) of the reference genome assembly of *L. sativa* cv. Salinas. (.docx, 121 KB)
- Figure S5 - Graphical representation of the third major resistance cluster on chromosome 8 (MRC8C) of the reference genome assembly of *L. sativa* cv. Salinas. (.docx, 235 KB)
- Figure S6 - Graphical representation of the major resistance cluster on chromosome 9 (MRC9A) of the reference genome assembly of *L. sativa* cv. Salinas. (.docx, 548 KB)
- Table S1 - Primers used for cloning fragments of candidate NLR-encoding genes into the RNAi vector and for transgene detection. (.docx, 21 KB)
- Table S2  - Genes potentially involved in pathogen recognition in lettuce grouped in chromosomal order with their identified domains, *RGC* family assignation, genomic location, scaffold coordinates, and previous nomenclature. (.xlsx, 165 KB)
- Table S3 - List of all the predicted *RGC*s by each of the constructs designed and tested for MRC8A. (.docx, 18 KB)
- Table S4 - Predicted RNAi targets for LEO266\_TIR\_RNAi, the only construct in MRC8B tested that targets *RGC4*s, the *RGC*s that co-segregate with the phenotype. (.docx, 19 KB)
- Table S5 - Predicted RNAi targets for the two constructs silencing members of the *RGC21* family. (.docx, 21 KB)
