# Supplementary material for: Genome-Wide Architecture of Disease Resistance Genes in Lettuce
Source: G3 (Bethesda). 2015 Oct 8;5(12):2655–69. doi: 10.1534/g3.115.020818 (PMC4683639; doi:10.1534/g3.115.020818)
Supplement: Supporting Information [file supp_g3.115.020818_FileS1.docx]

**File S1**

**Supporting Materials and Methods**

The backbone of pSmeagol was derived from the binary vector pGSA1165 (<http://chromdb.org/>) after restriction digestion with *Nco*I and *BamH*I to remove the *GUS* fragment. The vector backbone was ligated to a cassette containing a 300 bp *UidA* fragment in the antisense orientation, followed by an 800 bp *PDK* sequence and the same 300 bp *UidA* fragment in the sense orientation using a T4 DNA ligase (NEB). The new vector was verified by Sanger sequencing and was used to generate the RNAi constructs in the same manner as described previously (Wroblewski *et al.* 2007) with *Dra*III restriction sites introduced at the ends of the *UidA* sequences to permit ligation of the gene specific sequences and *Sfi*I restriction sites were added to the primers (Table S1) used for amplification of the gene fragments to facilitate directional cloning as detailed previously (Wroblewski *et al.* 2014). This resulted in the trigger sequence between a 300 bp fragment of the *UidA* reporter gene and the 35S promoter or *Nos* terminator in an inverted repeat structure (Figure S1). Each of the constructs was checked by digestion with *Bgl*II (NEB). There are two unique *Bgl*II restriction sites, one between the 35S promoter and the *UidA* antisense fragment and the other on the *PDK* fragment. The size of the smaller band would be ~1,681 bp plus the size of the gene specific fragment, approximately 400 bp greater than when the antisense orientation of the gene sequence is fused to the antisense *UidA* fragment (Figure S1).
