# Supplementary figures and images for: Genome-Wide Architecture of Disease Resistance Genes in Lettuce
Source: G3 (Bethesda). 2015 Oct 8;5(12):2655–69. doi: 10.1534/g3.115.020818 (PMC4683639; doi:10.1534/g3.115.020818)

**
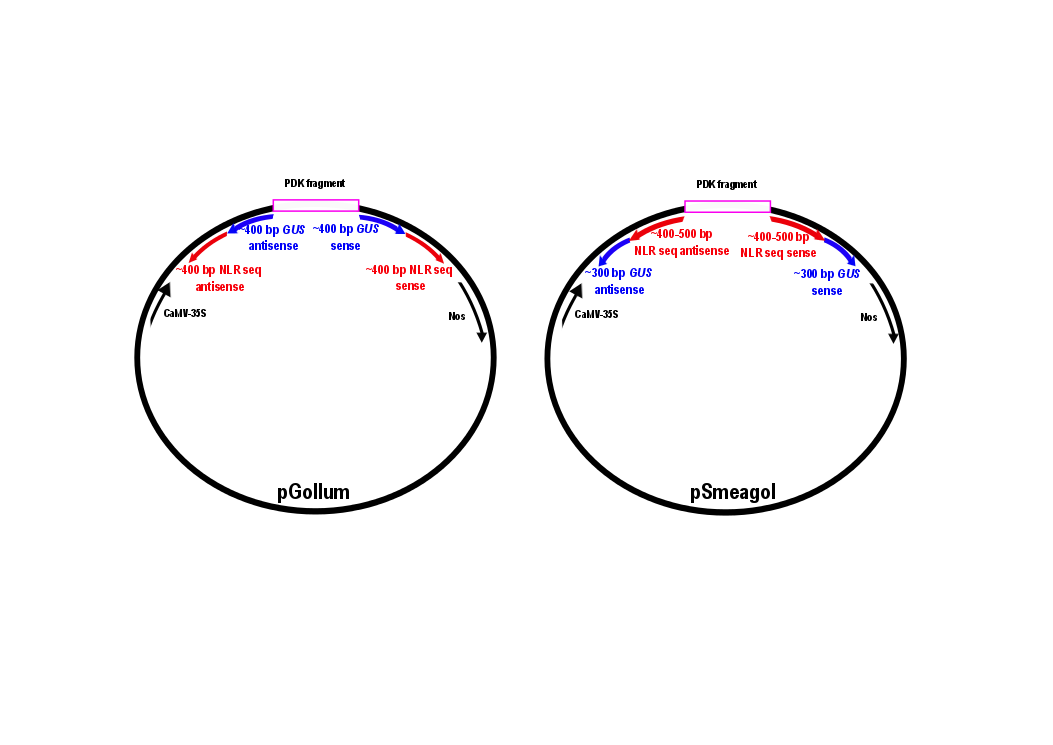
**

**Figure S1** Vectors used for RNAi constructs.

Supplement: Supporting Information [file supp_g3.115.020818_FigureS1.docx]
