# Supplementary material for: Genome-Wide Architecture of Disease Resistance Genes in Lettuce
Source: G3 (Bethesda). 2015 Oct 8;5(12):2655–69. doi: 10.1534/g3.115.020818 (PMC4683639; doi:10.1534/g3.115.020818)
Supplement: Supporting Information [file supp_g3.115.020818_FigureS3.docx]

**
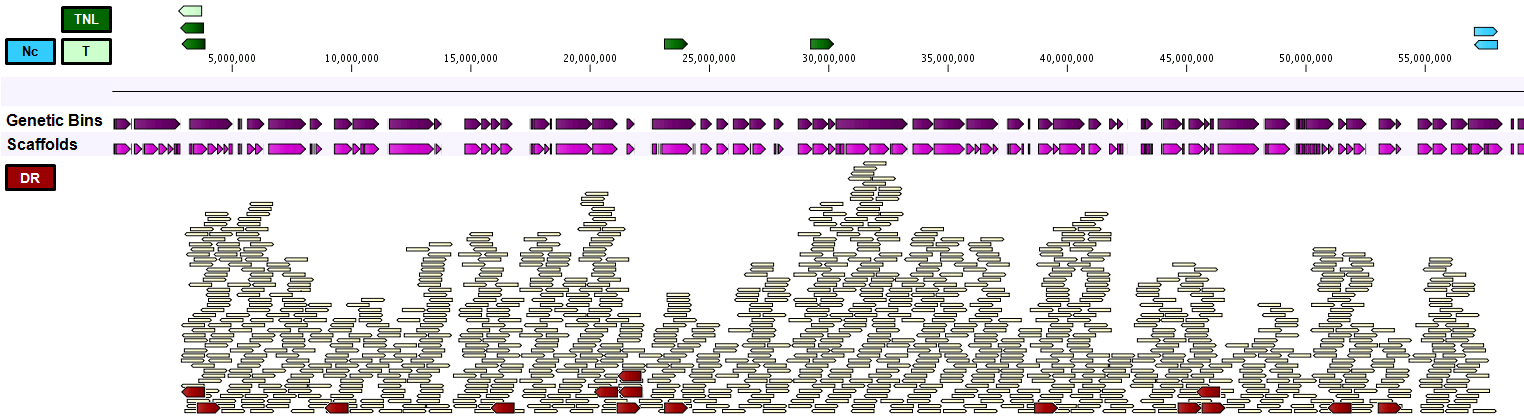
**

**Figure S3** Graphical representation of the first major resistance cluster on chromosome 8 (MRC8A) of the reference genome assembly of *L. sativa* cv. Salinas. The top panel shows the position of NLR encoding genes relative to the genetic bins and the scaffolds, whereas the bottom panel shows the position of all other genes that map to this locus. Color-coding: TNLs are colored in shades of green and CNLs in shades of blue, Defense Response (DR) genes in red and the remainder of the genes in yellow. The NLRs are further subdivided based on the number of characteristic domains detected. Abbreviations used: N for NB, L for LRR, C for CC, T for TIR. Lower case letters used whenever the domain itself was not detected but inferred based on the phylogeny of the NB domain.
