# Supplementary material for: Genome-Wide Architecture of Disease Resistance Genes in Lettuce
Source: G3 (Bethesda). 2015 Oct 8;5(12):2655–69. doi: 10.1534/g3.115.020818 (PMC4683639; doi:10.1534/g3.115.020818)
Supplement: Supporting Information [file supp_g3.115.020818_TableS1.docx]

**Table S1** Primers used for cloning fragments of candidate NLR-encoding genes into the RNAi vector and for transgene detection.

| **Target sequence** | **Oligonucleotide name** | **Oligonucleotide sequence** |
| --- | --- | --- |
| *Generation of RNAi constructs* | | |
| QGC20G02.yg.ab1 | QGC20G02_LRR_RNAi _L | CTGGCCTGAGCGGCCATTCTTTCTCCGGACCCATC |
| QGC20G02.yg.ab1 | QGC20G02_LRR_RNAi _R | CAGGCCGTGTAGGCCCCAGTCCCGGTGATATTCTG |
| QGD13H21.yg.ab1 | QGD13H21_NB_RNAi_L | CTGGCCTGAGCGGCCTGGAAGGGACAATTCCAAGA |
| QGD13H21.yg.ab1 | QGD13H21_NB_RNAi _R | CAGGCCGTGTAGGCCCCTTTTTACAGGCTGCTGCT |
| QGD7B12.yg.ab1 | QGD7B12_LRR_RNAi_L | ATGGCCATGTAGGCCACTCGATGATTTGGCTACCG |
| QGD7B12.yg.ab1 | QGD7B12_LRR__RNAi_R | ATGGCCAGAGAGGCCCAACTTCCCCAACAATGCTT |
| CLV_S1_Contig142 | Contig142_NB_RNAi_L | ATGGCCAGAGAGGCCGAAAGTGGGAACTCCTCCAA |
| CLV_S1_Contig142 | Contig142_NB_RNAi_R | ATGGCCATGTAGGCCATGCAAACAACTGCTTCAGG |
| QGC12K15.yg.ab1 | QGC12K15_TIR RNAi_L | ATGGCCATGTAGGCCCCTGATTTGGAGAGCGTGAT |
| QGC12K15.yg.ab1 | QGC12K15_TIR RNAi_R | ATGGCCAGAGAGGCCTCGGCCATTTACATCCTTTC |
| QGD6G21.yg.ab1 | QGD6G21_NB_RNAi_L | ATGGCCAGAGAGGCCCCATTCAAGTTCGTTATGTTTCT |
| QGD6G21.yg.ab1 | QGD6G21_NB_RNAi_R | ATGGCCATGTAGGCCCCTGTCTATATACCCTTCCCAAT |
| CLRX1526.b2_L22.ab1 | CLRX1526_LRR_RNAi_L | ATGGCCAGAGAGGCCTGCTTGAGGTTTGCGAAT |
| CLRX1526.b2_L22.ab1 | CLRX1526_LRR_RNAi_R | ATGGCCATGTAGGCCATGGAAGCGATCGGATTT |
| CLRX7678.b1_L24.ab1 | CLRX7678_NB_RNAi_L | ATGGCCAGAGAGGCCTATTGGTGGGATTGGGAAGA |
| CLRX7678.b1_L24.ab1 | CLRX7678_NB_RNAi_R | ATGGCCATGTAGGCCATGCTTGGGTTTAACGTTCG |
| CLSX3769.b1_B08.ab1 | CLSX3769_NB_RNAi_L | ATGGCCATGTAGGCCGCTCGGTTCAAGTTCGTGAT |
| CLSX3769.b1_B08.ab1 | CLSX3769_NB_RNAi_R | ATGGCCAGAGAGGCCAGCCATTGGATGTCCTTCAC |
| CLSM10181.b1_I02.ab1 | CLSM10181_NB_RNAi_L | ATGGCCAGAGAGGCCGAGTTGGATGCTGCAAGACA |
| CLSM10181.b1_I02.ab1 | CLSM10181_NB_RNAi_R | ATGGCCATGTAGGCCGACGAGGGAAGCATTGGATA |
| GB_EU889316.1 | LserNBS03_NB_L | ATGGCCAGAGAGGCCCCACAGCAGAACCAACTCCT |
| GB_EU889316.1 | LserNBS03_NB_R | ATGGCCATGTAGGCCTCCCTGCAACGTCTATCACA |
| CLX_S3_Contig1306 | Contig_1306_NB_RNAi_L | ATGGCCAGAGAGGCCCATCGGAACACAGTGACACC |
| CLX_S3_Contig1306 | Contig_1306_NB_RNAi_R | ATGGCCATGTAGGCCAATCTGCTGTTGGCACCTCT |
| CLS_S3_Contig7390 | Contig_7390_LRR_RNAi_L | ATGGCCAGAGAGGCCGTGGTTCCACAGGACGAAGT |
| CLS_S3_Contig7390 | Contig_7390_LRR_RNAi_R | ATGGCCATGTAGGCCGGAATCTTGCCACCAAGGTA |
| GB_EU889302.1 | LsatNBS09_NB_RNAi_L | ATGGCCAGAGAGGCCTGTAACTGCTAACGGGAGACC |
| GB_EU889302.1 | LsatNBS09_NB_RNAi_R | ATGGCCATGTAGGCCTGATGGTTGTTGCTTTGTTGA |
| QGC7A16.yg.ab1 | QGC7A16_LRR_RNAi_L | ATGGCCAGAGAGGCCGTGGGAATCAGAAGCAGAGG |
| QGC7A16.yg.ab1 | QGC7A16_LRR_RNAi_R | ATGGCCATGTAGGCCTCATGGTCAGGAAATGACTCC |
| GB_AF017754.1 | AF017754_NB_RNAi_L | ATGGCCAGAGAGGCCCCAGGAAGTTTGATGGCTGT |
| GB_AF017754.1 | AF017754_NB_RNAi_R | ATGGCCATGTAGGCCACCAGCATAAGCAACCACGTC |
| GB_EU889315.1 | LserNBS02_NB_RNAi_L | ATGGCCAGAGAGGCCAACGATTTGTCATTAGGAGCA |
| GB_EU889315.1 | LserNBS02_NB_RNAi_R | ATGGCCATGTAGGCCGCATTGGGTGTGGATAACTTT |
| GB_AY153833.1 | AY153833.1_LRR_RNAi_L | ATGGCCAGAGAGGCCGTTGCATCGCCATGAATTTG |
| GB_AY153833.1 | AY153833.1_LRR_RNAi_R | ATGGCCATGTAGGCCTTGCTCCAATCAACTCATCC |
| QGG13M01.yg.ab1 | LE0395_LRR_RNAi_L | ATGGCCATGTAGGCCGGCAACCAAGAAACAGAACC |
| QGG13M01.yg.ab1 | LE0395_LRR_RNAi_R | ATGGCCAGAGAGGCCCCAACAGCCTTAAGTTGGTC |
| GB_EU889303.1 | Lsat11_NB_RNAi_L | ATGGCCATGTAGGCCAGGCTCCTTTGGTGATTCCT |
| GB_EU889303.1 | Lsat11_NB_RNAi_R | ATGGCCAGAGAGGCCGAAGATACAATGCGCCAGGT |
| CLX_S3_Contig5632 | Contig5632_TIR_RNAi_L | ATGGCCAGAGAGGCCATGAGAGGGCAAATCGTGA |
| CLX_S3_Contig5632 | Contig5632_TIR_RNAi_R | ATGGCCATGTAGGCCGCAATAAAGCAACAACCATCAA |
| QGD14O14.yg.ab1 | QGD14O14_NB_RNAi_L | ATGGCCAGAGAGGCCAGTAAGCTTGGCGTTCATGG |
| QGD14O14.yg.ab1 | QGD14O14_NB_RNAi_R | ATGGCCATGTAGGCCCTTCCATGAGGCGAGTTGTAG |
| GB_AY153836.1 | AY153836_NB_RNAi_L | CTGGCCTGAGCGGCCAGCCAAACATGTCTATGGGTTA |
| GB_AY153836.1 | AY153836_NB_RNAi_R | CAGGCCGTGTAGGCCGGTTCCAAGTAATGCATCCAA |
| QG_CA_Contig946 | LEO414_LRR_RNAi_L | CTGGCCTGAGCGGCCGAAGACGGATTTGGTGCATT |
| QG_CA_Contig946 | LEO414_LRR_RNAi_R | CAGGCCGTGTAGGCCACGCAACGGCAATGAATACT |
| QGH8M10.yg.ab1 | LEO266_TIR_RNAi_L | CTGGCCTGAGCGGCCTGCAGATTCATCGTGGTGTT |
| QGH8M10.yg.ab1 | LEO266_TIR_RNAi_R | CAGGCCGTGTAGGCCTGTTGGCAATAAACCATCCA |
|  | LsatNBS05_NB_RNAi_L | ATGGCCAGAGAGGCCTCCCAGTATGGCCTCTCTTC |
|  | LsatNBS05_NB_RNAi_R | ATGGCCATGTAGGCCGCTAAAGTCGGTTCTACAATGATAAA |
|  | RGC2B_NB_RNAi_L | AGAATGATGCAAAGGCTGAAGAAGG |
|  | RGC2B_NB_RNAi_R | GAAAGTGCATCCTTCCATGCATC |
| *Detection of RNAi construct* | | |
| One arm of inverted repeats | TransgeneF | GGATTGATTACAGTTGGG |
| One arm of inverted repeats | TransgeneL | GCAGGACTCTAGGGACTAG |
| *Genomic control* | | |
|  | Le9005_F | GGTAACACAGGAACACGATGAGCT |
|  | Le9005_R | AGAGGCAACATCACCAAATCCTTC |
