# Supplementary material for: Genome-Wide Architecture of Disease Resistance Genes in Lettuce
Source: G3 (Bethesda). 2015 Oct 8;5(12):2655–69. doi: 10.1534/g3.115.020818 (PMC4683639; doi:10.1534/g3.115.020818)
Supplement: Supporting Information [file supp_g3.115.020818_TableS3.docx]

Table S3 List of all the predicted *RGCs* by each of the constructs designed and tested for MRC8A.

| Old ID | New ID | RGC family | RNAi construct | Identity |
| --- | --- | --- | --- | --- |
| Lsa022918.1 | 8_1861.1 | *RGC4* | Lsat11_NB_RNAi | NT^1^ |
| Lsa036920.1 | 8_2021.1 | *RGC4* | Lsat11_NB_RNAi | NT |
| Lsa022947.1 | 8_14700.1 | *RGC4* | Lsat11_NB_RNAi | NT |
| Lsa006288.1 | 8_18160.1 | *RGC4* | Lsat11_NB_RNAi | NT |
| Lsa026991.1 | 8_35940.1 | *RGC27* | Lsat11_NB_RNAi | NT |
| Lsa026992.1 | 8_36021.1 | *RGC27* | Lsat11_NB_RNAi | NT |
| Lsa022918.1 | 8_1861.1 | *RGC4* | Contig5632_TIR_RNAi | at least 4 x 21nt |
| Lsa036920.1 | 8_2021.1 | *RGC4* | Contig5632_TIR_RNAi | at least 4 x 21nt |
| Lsa022947.1 | 8_14700.1 | *RGC4* | Contig5632_TIR_RNAi | at least 2 x 21nt |
| Lsa006288.1 | 8_18160.1 | *RGC4* | Contig5632_TIR_RNAi | NT |
| Lsa026991.1 | 8_35940.1 | *RGC27* | Contig5632_TIR_RNAi | NT |
| Lsa026992.1 | 8_36021.1 | *RGC27* | Contig5632_TIR_RNAi | NT |
| Lsa022918.1 | 8_1861.1 | *RGC4* | QGD14O14_NB_RNAi | NT |
| Lsa036920.1 | 8_2021.1 | *RGC4* | QGD14O14_NB_RNAi | NT |
| Lsa022947.1 | 8_14700.1 | *RGC4* | QGD14O14_NB_RNAi | NT |
| Lsa006288.1 | 8_18160.1 | *RGC4* | QGD14O14_NB_RNAi | NT |
| Lsa026991.1 | 8_35940.1 | *RGC27* | QGD14O14_NB_RNAi | NT |
| Lsa026992.1 | 8_36021.1 | *RGC27* | QGD14O14_NB_RNAi | NT |

^1^Not predicted target.
