# Supplementary material for: Genome-Wide Architecture of Disease Resistance Genes in Lettuce
Source: G3 (Bethesda). 2015 Oct 8;5(12):2655–69. doi: 10.1534/g3.115.020818 (PMC4683639; doi:10.1534/g3.115.020818)
Supplement: Supporting Information [file supp_g3.115.020818_TableS4.docx]

**Table S4** Predicted RNAi targets for LEO266_TIR_RNAi, the only construct in MRC8B tested that targets *RGC4*s, the *RGC*s that co-segregate with the phenotype. The constructs AY153833.1_LRR_RNAi (*RGC15*), LEO_395_LRR_RNAi (*RGC29*), LEO414_LRR_RNAi (*RGC9*) also tested for the MRC8B phenotype target members of the gene family they belong to but no *RGC4* genes.

| Old ID | New ID | RGC family | Identity |
| --- | --- | --- | --- |
| Lsa026205.1 | 8_47121.1 | *RGC4* | at least 1 x 21nt |
| Lsa026204.1 | 8_47141.1 | *RGC4* | no |
| Lsa077712.1 | 8_47161.1 | *RGC4* | no |
| Lsa026222.1 | 8_47261.1 | *RGC4* | no |
| Lsa026220.1 | 8_47281.1 | *RGC4* | no |
| Lsa026219.1 | 8_47361.1 | *RGC4* | no |
| Lsa077136.1 | 8_48221.1 | *RGC4* | no |
| Lsa017362.1 | 8_48241.1 | *RGC4* | 97% identical |
| Lsa077138.1 | 8_48341.1 | *RGC4* | no |
| Lsa017359.1 | 8_48361.1 | *RGC4* | no |
| Lsa036549.1 | 8_48440.1 | *RGC4* | at least 1 x 21nt |
| Lsa010255.1 | 8_48581.1 | *RGC4* | no |
| Lsa017005.1 | 8_48621.1 | *RGC4* | no |
| Lsa034515.1 | 8_48660.1 | *RGC4* | no |
| Lsa034519.1 | 8_48680.1 | *RGC4* | no |
| Lsa034516.1 | 8_48700.1 | *RGC4* | no |
| Lsa034514.1 | 8_48740.1 | *RGC4* | no |
| Lsa034517.1 | 8_48760.1 | *RGC4* | no |
| Lsa014954.1 | 8_48960.1 | *RGC4* | no |
| Lsa014945.1 | 8_49000.1 | *RGC4* | no |
| Lsa022849.1 | 8_49401.1 | *RGC4* | no |
| Lsa022847.1 | 8_49481.1 | *RGC4* | no |
| Lsa034187.1 | 8_49780.1 | *RGC4* | at least 1 x 21nt |
| Lsa034186.1 | 8_49880.1 | *RGC4* | no |
| Lsa025050.3 | 8_49901.1 | *RGC4* | no |
| Lsa025048.3 | 8_49961.1 | *RGC4* | no |
| Lsa025049.1 | 8_49981.1 | *RGC4* | no |
| Lsa041293.1 | 8_50060.1 | *RGC4* | no |
| Lsa001849.1 | 8_50380.1 | *RGC4* | no |
| Lsa001840.1 | 8_50421.1 | *RGC4* | no |
| Lsa001846.1 | 8_50481.1 | *RGC4* | no |
| Lsa001844.1 | 8_50541.1 | *RGC4* | no |
| Lsa001285.1 | 8_50621.1 | *RGC4* | no |
| Lsa001284.1 | 8_50681.1 | *RGC4* | no |
| Lsa001286.1 | 8_50701.1 | *RGC4* | at least 1 x 21nt |
| Lsa019175.1 | 8_51501.1 | *RGC4* | at least 1 x 21nt |
