# Supplementary material for: Genome-Wide Architecture of Disease Resistance Genes in Lettuce
Source: G3 (Bethesda). 2015 Oct 8;5(12):2655–69. doi: 10.1534/g3.115.020818 (PMC4683639; doi:10.1534/g3.115.020818)
Supplement: Supporting Information [file supp_g3.115.020818_TableS5.docx]

**Table S5 Predicted RNAi targets for the two constructs silencing members of the *RGC21* family**. The QGC7A16_LRR_RNAi construct abrogated the *Dm13-*mediated resistance while the LserNBS02_NB_RNAi construct did not.

| Targeted Gene ID | Genome position | RGC family | LRR RNAi Construct | Identity | NB RNAi Construct | Identity1 |
| --- | --- | --- | --- | --- | --- | --- |
| 3_118460.1 | chr3 | *RGC21* | QGC7A16 | almost perfect match | LserNBS02 | at least 2 x 21 |
| 3_118201.1 | chr3 | *RGC21* | QGC7A16 | perfect match | LserNBS02 | at least 10 x 21 |
| 3_120821.1 | chr3 | *RGC21* | QGC7A16 | at least 4 x 21 | LserNBS02 | at least 3 x 21 |
| 3_118260.1 | chr3 | *RGC21* | QGC7A16 | at least 2 x 21 | LserNBS02 | at least 1 x 21 |
| 0_19880.1 | not mapped | *RGC21* | QGC7A16 | at least 2 x 21 | LserNBS02 | at least 1 x 21 |
| 0_30981.1 | not mapped | *RGC21* | QGC7A16 | at least 2 x 21 | LserNBS02 | at least 1 x 21 |
| 3_117761.1 | chr3 | *RGC21* | QGC7A16 | at least 2 x 21 | LserNBS02 | at least 2 x 21 |
| 0_19860.1 | not mapped | *RGC21* | QGC7A16 | at least 2 x 21 | LserNBS02 | at least 2 x 21 |
| 0_19660.1 | not mapped | *RGC21* | QGC7A16 | at least 1 x 21 | LserNBS02 | at least 1 x 21 |
| 3_116920.1 | chr3 | *RGC21* | QGC7A16 | at least 1 x 21 | LserNBS02 | at least 1 x 21 |
| 3_116500.1 | chr3 | *RGC21* | QGC7A16 | at least 1 x 21 | LserNBS02 | at least 1 x 21 |
| 3_118060.1 | chr3 | *RGC21* | QGC7A16 | at least 1 x 21 | LserNBS02 | at least 2 x 21 |
| 3_118161.1 | not mapped | *RGC21* | QGC7A16 | at least 4 x 21 | LserNBS02 | at least 6 x 21 |
| 3_120901.1 | chr3 | *RGC21* | QGC7A16 | at least 4 x 21 | LserNBS02 | almost perfect match |
| 3_120901.1 | chr3 | *RGC21* | QGC7A16 | at least 2 x 21 | LserNBS02 | almost perfect match |
| 3_120701.1 | chr3 | *RGC21* | QGC7A16 | no 1 x21 | LserNBS02 | at least 3 x 21 |
| 3_117880.1 | chr3 | *RGC21* | QGC7A16 | no 1 x21 | LserNBS02 | at least 3 x 21 |
| 3_118121.1 | chr3 | *RGC21* | QGC7A16 | no 1 x21 | LserNBS02 | at least 2 x 21 |
| 3_115800.1 | chr3 | *RGC21* | QGC7A16 | no 1 x21 | LserNBS02 | at least 1 x 21 |
| 3_113940.1 | chr3 | *RGC21* | QGC7A16 | no 1 x21 | LserNBS02 | at least 1 x 21 |
| 5_164620.1 | chr5 | *RGC1* | QGC7A16 | no 1 x21 | LserNBS02 | at least 1 x 21 |
| 7_76440.1 | chr7 | *RGC1* | QGC7A16 | no 1 x21 | LserNBS02 | at least 1 x 21 |
| 1_101001.1 | chr1 | *RGC1* | QGC7A16 | no 1 x21 | LserNBS02 | at least 1 x 21 |
| 3_117620.1 | chr3 | Na2 | QGC7A16 | at least 5 x 21 | LserNBS02 | no 1 x21 |
| 3_116540.1 | chr3 | Na | QGC7A16 | at least 2 x 21 | LserNBS02 | no 1 x21 |
| 3_116460.1 | chr3 | Na | QGC7A16 | at least 2 x 21 | LserNBS02 | no 1 x21 |
| 0_17700.1 | not mapped | Na | QGC7A16 | at least 2 x 21 | LserNBS02 | no 1 x21 |
| 0_43420.1 | not mapped | Na | QGC7A16 | at least 1 x 21 | LserNBS02 | at least 1 x 21 |
| 3_120741.1 | chr3 | Na | QGC7A16 | no 1 x21 | LserNBS02 | at least 4 x 21 |
| 0_19800.1 | not mapped | Na | QGC7A16 | no 1 x21 | LserNBS02 | at least 2 x 21 |

^1^Identity measured as fragments of > 20 nt with 100% sequence identity to the RNAi trigger sequence at the nucleotide level. Almost perfect match refers to the entire gene fragment cloned as inverted repeats into the RNAi vector.

^2^Na: not assigned to an *RGC* family due to the lack of an NB domain.
